# Survivability of the lichen *Xanthoria parietina* in simulated Martian environmental conditions

**DOI:** 10.1038/s41598-023-32008-6

**Published:** 2023-03-25

**Authors:** Christian Lorenz, Elisabetta Bianchi, Giovanni Poggiali, Giulia Alemanno, Renato Benesperi, John Robert Brucato, Stephen Garland, Jörn Helbert, Stefano Loppi, Andreas Lorek, Alessandro Maturilli, Alessio Papini, Jean-Pierre de Vera, Mickaël Baqué

**Affiliations:** 1 Department of Biology, University of Florence, Via la Pira 4, 50121 Florence, Italy; 2 LESIA-Observatoire de Paris, CNRS, Université PSL, Sorbonne Université, Université de Paris, 5 Place Jules Janssen, 92190 Meudon, France; 3 INAF-Astrophysical Observatory of Arcetri, Largo E. Fermi 5, 50125 Florence, Italy; 4 Planetary Laboratories Department, Institute of Planetary Research, German Aerospace Center (DLR), Ruthefordstraße 2, 12489 Berlin, Germany; 5 Department of Environmental Sciences, University of Siena, Via P. A. Mattioli 4, 53100 Siena, Italy; 6 Microgravity User Support Center (MUSC), Space Operations and Astronaut Training, German Aerospace Center (DLR), Linder Höhe, 51147 Cologne, Germany

**Keywords:** Photosystem II, Ecophysiology

## Abstract

*Xanthoria parietina* (L.) Th. Fr. is a widely spread foliose lichen showing high tolerance against UV-radiation thanks to parietin, a secondary lichen substance. We exposed samples of *X. parietina* under simulated Martian conditions for 30 days to explore its survivability. The lichen’s vitality was monitored via chlorophyll *a* fluorescence that gives an indication for active light reaction of photosynthesis, performing in situ and after-treatment analyses. Raman spectroscopy and TEM were used to evaluate carotenoid preservation and possible variations in the photobiont’s ultrastructure respectively. Significant differences in the photo-efficiency between UV irradiated samples and dark-kept samples were observed. Fluorescence values correlated with temperature and humidity day-night cycles. The photo-efficiency recovery showed that UV irradiation caused significant effects on the photosynthetic light reaction. Raman spectroscopy showed that the carotenoid signal from UV exposed samples decreased significantly after the exposure. TEM observations confirmed that UV exposed samples were the most affected by the treatment, showing chloroplastidial disorganization in photobionts’ cells. Overall, *X. parietina* was able to survive the simulated Mars conditions, and for this reason it may be considered as a candidate for space long-term space exposure and evaluations of the parietin photodegradability.

## Introduction

Two of the hot astrobiological topics proposed by the European Astrobiology Roadmap (AstRoMap project) are the study of the limits of life in simulated/real extreme environments and the study of particular biomolecules that could represent biomarker of present/past life-forms out of Earth’s biosphere^1^. Investigating the limits of life in stressful environments allows to explore the physiological and biochemical effects of extreme conditions on biological samples^2^. In the past thirty years biologists realized that very extreme and inhospitable habitats can support life^3^. The study of extremophiles, able to survive in critical conditions, and pioneer species, those colonizing environments first, was crucial in the development of astrobiology^4,5^. Among them, lichens have proved to thrive and survive in some of the most extreme habitats on Earth^3,6–10^. Therefore, the eco-physiology of these organisms might give an indication about their potential adaptive plasticity in the perspective of climate change, past (and future) terrestrial geological scenarios^11^ and extra-terrestrial habitats such as Mars’ surface and exoplanets.

Several studies proved lichens’ resistance when exposed to space and Mars-like conditions. Lichens’ high resistance to extreme conditions is due to their main eco-physiological features (poikilohydry and anhydrobiosis) and metabolic processes (production of the secondary lichen substances). The BIOPAN experiment showed that *Rusavskia elegans* (Link) S.Y. Kondr. & Kärnefelt (2003), *Rhizocarpon geographicum* (L.) DC. s.lat. and *Circinaria fruticulosa* (Eversm.) Sohrabi (2012) samples survived for 10–14 days in space, being metabolically active and able to grow after the exposure^8,12–17^. The LIFE experiment aimed to expose for 1.5 years different biological samples in space. *Rusavskia elegans* showed a high recovery in post-flight photosynthetic efficiency measurements compared to the other tested species^18^.

On the other hand, ground-based experiments allow us to simulate extreme conditions and to monitor the organisms’ viability parameters in situ or immediately after the treatment^3,6^. *Pleopsidium chlorophanum* (Wahlenb.) Zopf was tested in Mars niche condition at the Mars Simulation Facility of the German Aerospace Center (DLR Berlin, Germany)^20^. The results confirmed the lichen’s capability to physiologically adapt and increase photosynthesis during the 34 days of exposure to Mars niche conditions. Instead, *C. fruticulosa* was exposed physiologically active in both Mars niche and surface-like conditions for 30 days at the Mars Simulation Facility too^19^ and only the niche samples showed viability responses. In order to investigate hazardous ionizing radiation effects on lichens, desiccated *R. elegans* thalli were irradiated at the National Institute of Radiological Sciences (NIRS) in Chiba, Japan^21^. The results revealed a significant photoefficiency decrease but not correlated with the applied increasing doses, suggesting the high survival capacity of anhydrobiotic thalli.

In this study, the lichen species *Xanthoria parietina* (L.) Th. Fr. was exposed to simulated Mars-like conditions for 30 days at the Planetary Analogue Simulation LABoratory (PASLAB) at the DLR in Berlin. The species is a widespread foliose lichen growing on barks and rocks, colonizing almost all habitats from the seashore to the tree-line^22^. It grows in common and anthropized environments too, being tolerant to air pollutants^23^ such as NO_X_ and heavy metals^24^. *Xanthoria parietina* was chosen for this experiment because of its tolerance to UV-radiation due to the lichen substance parietin which protects the photobiont layer (algal partner in the lichen symbiosis) in addition to the mycobiont mucilage and hyphae matrix. Moreover, parietin protects the algal photosystem apparatus from high light influx^25^ and its production is stimulated by UVB radiation^26,27^. Specifically, *X. parietina* has been already exposed to simulated space conditions (up to 16 h of space vacuum at 10^−3^ Pa and UV-radiation 160–400 nm range) along with *Gyalolechia bracteata* (Hoffm.) A. Massal., *R. elegans* (a parietin-producing lichen present on sunlight exposed rocks over the tree-line) at the Planetary and Space Simulation facilities (DLR, Köln, Germany)^6^. After exposure, the ascospores’ germination rate in Mars-analog soil was as high as on the other mineral media. *Rusavskia elegans* ascospores showed the highest survivability and *X. parietina* showed a remarkable germination rate of the irradiated ascospores too. At INAF—Astrophysical Observatory of Arcetri (Florence), *X. parietina* was exposed in two extremely dehydrating conditions—UV-radiation in N_2_ atmosphere and UV-radiation in vacuum 10^0^–10^−2^ Pa (both 1.34 MJ m^−2^ final dose) and for the first time, in situ IR spectroscopic analyses were performed on the exposed lichen species^28^. The recovery capability was evaluated assessing chlorophyll *a* (Chl *a*) fluorescence, showing a full recovery in N_2_ exposed thalli and a 50% recovery in vacuum-exposed samples, after 72 h from the treatment^28^. As *X. parietina* was able to survive these conditions, it was considered an optimal candidate for further evaluations on its survival capacity in extreme conditions. Specifically, we exposed for the first time *X. parietina* in Mars simulated conditions, characterized by a 95% CO_2_ atmosphere at 600 Pa, with a day-night cycle of relative humidity (0.1–100%) and temperature (16–55 °C), and UV-irradiation.

Our aims were to investigate *X. parietina*’s survivability in the overmentioned conditions and to study its recovery capacity, performing for the first time a multi-analyses approach with a before-after-control-impact protocol. The main purposes of the study were (i) to evaluate in situ Chl *a* fluorescence as an indicator of the active light reaction of photosynthesis, through real-time monitoring of lichen viability during the exposure to Mars-like conditions and after them during the recovery period; (ii) to assess significant changes in the peaks’ features of carotenoids in the specimens’ Raman spectra and (iii) to identify eventual ultrastructural changes in the algae cells through transmission electron microscopy.

## Material and methods

### Lichen material

**Figure 1:**
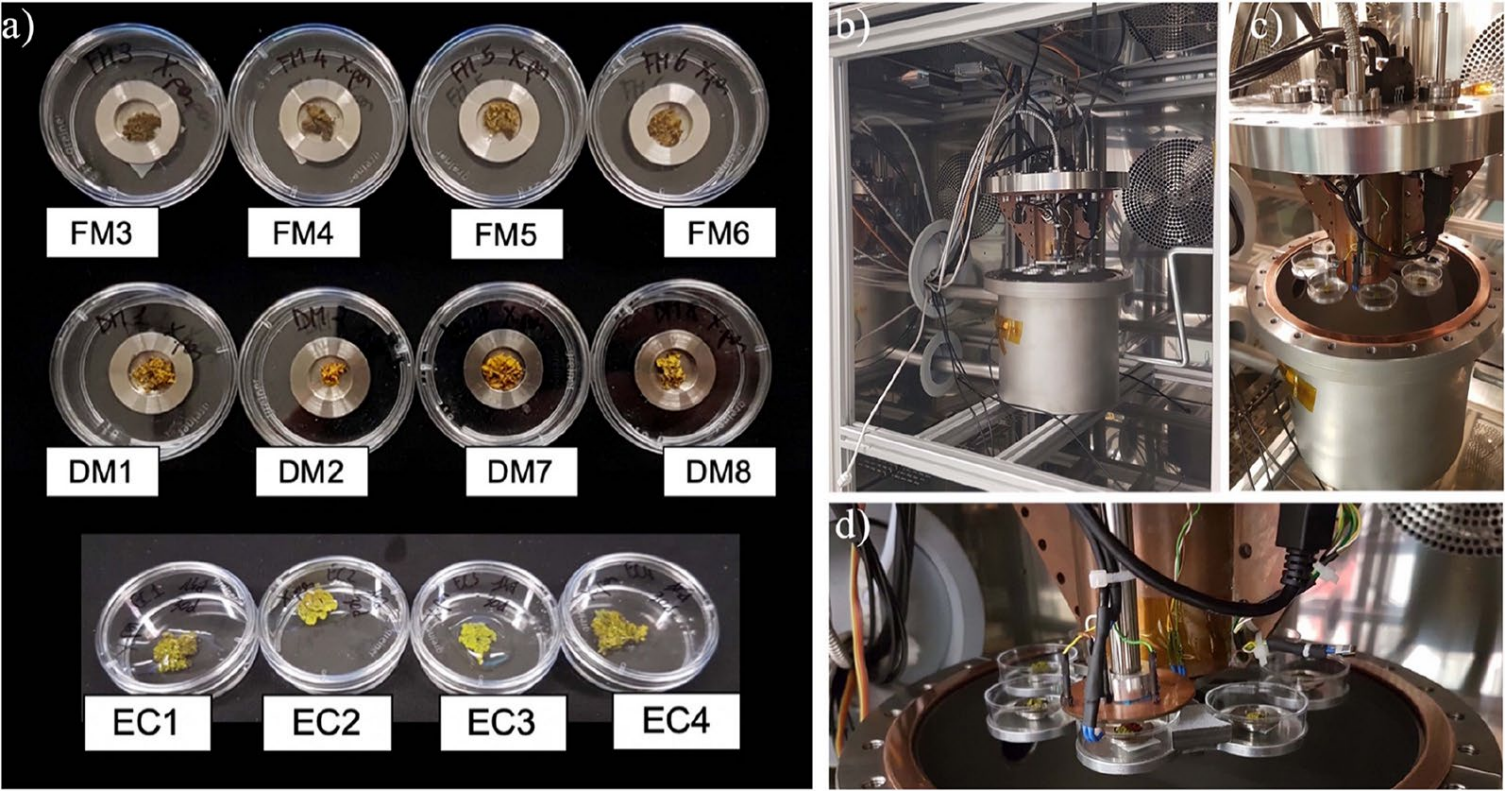
On the left (**a**), samples of *X. parietina* used in this experiment before the 30 days exposure. First row: Full Mars (FM) samples; second row: Dark Mars (DM) samples; third row: External Control (EC) samples. On the right, PASLAB at Berlin DLR. (**b**) Opened climate chamber and opened experiment chamber. (**c**) Detail of the opened experiment chamber with the sample holders on the turntable. (**d**) Detail on the sample position 1 with the above Mini PAM optical fiber.

Twelve thalli of *X. parietina* were randomly collected in a remote area of Florence province (Tuscany, Italy 43°59′25.09″ N 11°13′24.65″ E, at ca. 300 m a.s.l.) in August 2021. Specimens were dehydrated for 24 h at room temperature (25 °C) and stored at − 18 °C until treatment, as this procedure ensures that thalli remain healthy for later experiments and physiological analyses^29^. Three days before the pre-exposure analyses, specimens were allowed to slowly recover their normal metabolic conditions in a growth chamber at 25 °C, with 12 h dark and 12 h light at 50 μmol m^-2^ s^−1^ PAR photons, daily sprayed with distilled water^28,30,31^. On the third day, photoefficiency measurements were performed to assess proper thalli reactivation. They were dehydrated for 24 h and cut to obtain 12 samples of 1 cm^2^ in size for the next analyses^28^. The size was determined by the aluminum sample holders adopted for the exposure (Fig. 1a).

### Planetary analogue simulation laboratory

#### Description of the experiment equipment

**Figure 2:**
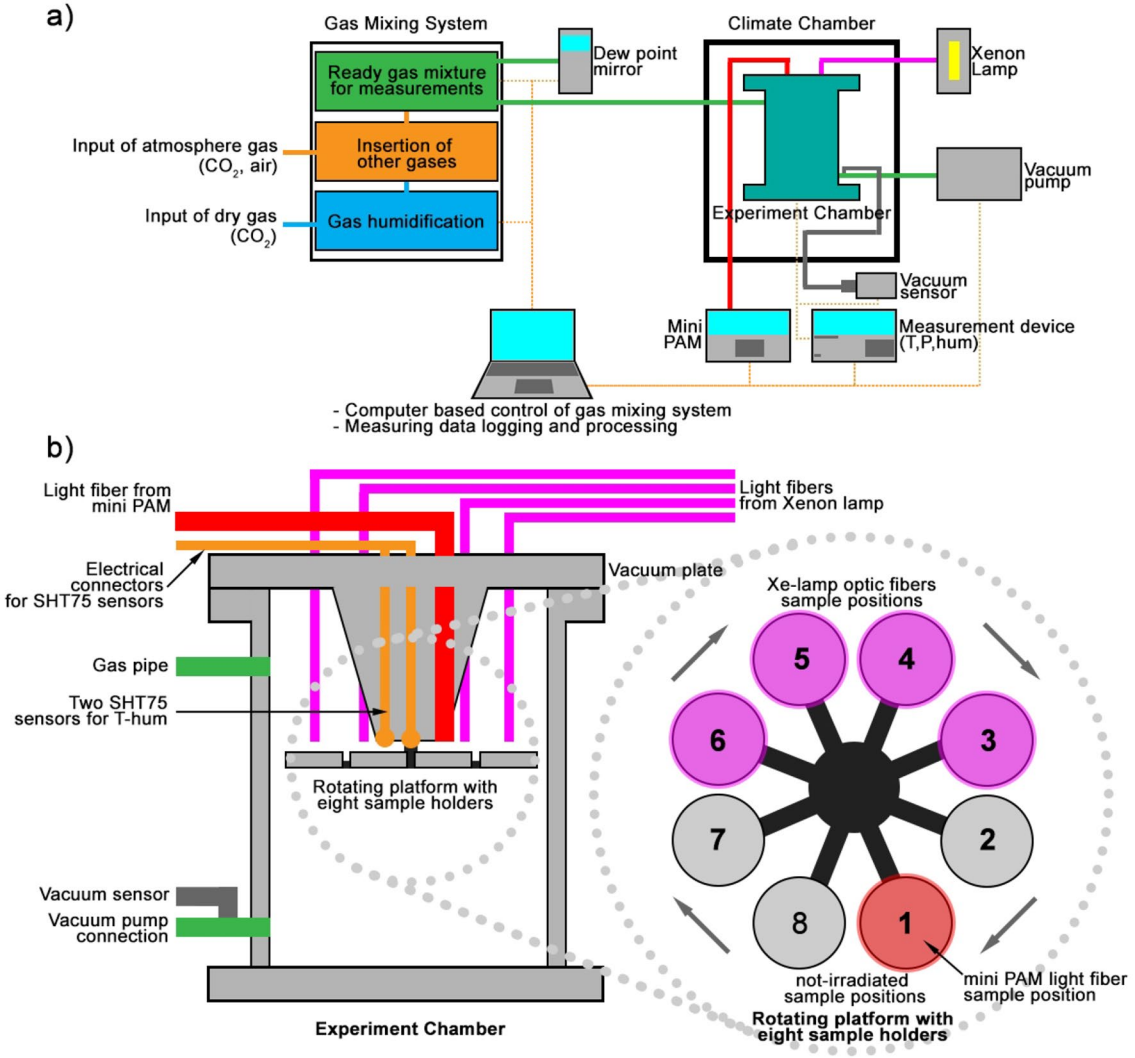
(**a**) Experiment configuration of the PASLAB facility. (**b**) On the left, experiment arrangement inside the experiment chamber, showing UV Xe-lamp optical fibers and Mini PAM light fiber connections. Gas connections are shown too. On the right, above vision on the turntable/rotating platform with the eight sample holders.

The experiment was carried out at the PASLAB of the Berlin DLR (Figs. 1, 2). The main focus of the PASLAB is the Mars Simulation Facility^32^, employing an ACS Discovery My DM340(C) climate chamber (ATT Umweltsimulation GmbH) with a temperature range from 180 to − 75 °C. The climate chamber’s internal dimensions are of 601 mm width, 810 mm depth and 694 mm height (Fig. 1b). The experiment was performed in a vacuum sealed stainless steel vessel with a volume of 10.3 L, an inner diameter of 20 cm and a height of 32 cm (Fig. 1c). The experimental chamber has electrical connectors, inlet/outlet gas connectors, four optical fibers for UV light, and one fiber for photosynthetic activity measurements obtained with a photosynthesis yield analyzer (Mini PAM, Walz GmbH, Germany)^32^. The UV source is a 150 W Xenon lamp interfaced to the four fibers. The light is focused using adjustable lenses onto four sample spots (Fig. 2). The radiation dose of the Xe-UV light is measured with an X92-optometer and an RCH-106-4 probe (Gigahertz-Optik GmbH, Germany) at wavelengths ranging from 250 to 400 nm^19^. Inside the chamber, there is a rotating platform with eight aluminum sample holders, four of them UV-irradiated and four of them in dark conditions^19,32^ (Figs. 1d, 2b). The Mini PAM optical fiber is located on the first sample position (Fig. 2b). Additionally, the experiment chamber is equipped with two SHT75 sensors (Sensirion AG, Switzerland) integrated with two Pt-100 temperature sensors to measure humidity and temperature close to the turntable. The humidity sensors have been calibrated for the Martian atmosphere^32^. The gas flow through the experiment chamber is generated by a gas mixing system, which can control up to five gases and allows precise control of the gas humidity via a humidification system for CO_2_ (Fig. 2a). The inside pressure is controlled by a membrane vacuum pump (MV10Vario, Vacuubrand GmbH)^32^. The control of the whole system is based on a DAQ-system device (National Instruments Corp., US) and Labview software. Figure 2 shows a scheme of the entire system.

#### Experimental conditions

**Figure 3:**
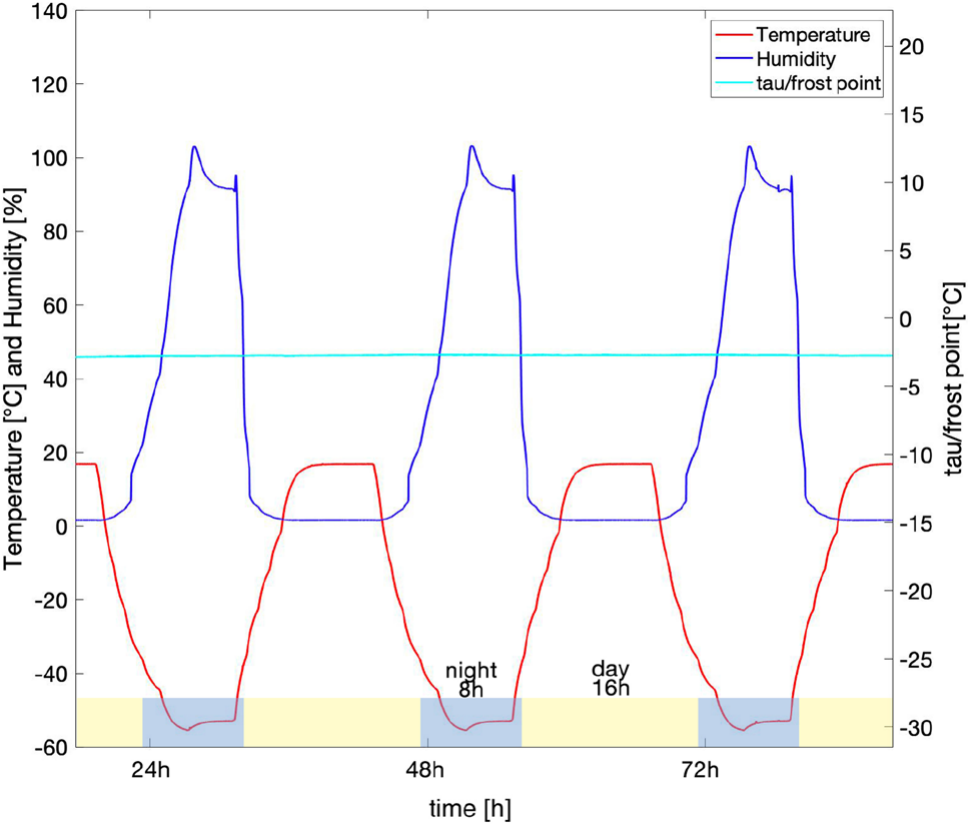
An instance over three days of the Mars-like diurnal cycle profile of temperature (red line) and relative humidity (blue line). The cyan line indicates the frost point temperature.

The PASLAB is part of the Department of Planetary Laboratories and the facility is used to perform laboratory experiments with controlled time variation (e.g. simulated diurnal variations) of temperature and humidity. Atmospheric pressure and composition can be set to simulate Mars-like conditions. In particular, the climate chamber can be set to the thermo-physical conditions typical of Martian mid/low latitudes^20^. During a complete experimental run the experiment chamber had a continuous gas flow of 20 l h^−1^ (at atmospheric pressure). The dry gas mixture contained 95% CO_2_, and 5% air (4% N_2_ and 1% O_2_). The pressure inside the experiment chamber was approximately 600 Pa, the temperature varied diurnally between 16 (day) and − 55 °C (night), whereas the relative humidity (with respect to ice) ranged between approximately 0.1% (day) and 100% (night). The experimental conditions are summarized in Table 1 and Fig. 3. The humidity of the gas flow was approximately − 2.8 ± 0.2 °C frost point temperature (tau/frost point, Fig. 3) at 101,325 Pa over the entire experiment, which corresponds to − 52.6 °C at 600 Pa^20^.

**Table 1:** Experimental conditions used at the PASLAB of the DLR Institute of Planetary Research in Berlin compared to Mars surface conditions.

Range of experimental and environmental parameters	Mars simulated conditions at the PASLAB	Mars conditions
Relative humidity	0.1% (day)–100% (night)^19,20,32^	0–100%^35,36^
Pressure	600 Pa^7,32^	680–790 Pa^35–38^
Temperature	from 16 °C (day) to − 55 °C (night), simulation of equatorial latitudes^19,20,32^	Mean value − 55 °C, − 130 °C at the poles to 27 °C at the equatorial regions^35,36,39,40^
Atmospheric gas composition	95% CO_2_, 4% N_2_ and 1% O_2_^9,20,32^	95% CO_2_, 2.8% N_2_, 2% Ar, 0.174% O_2_ and 0.0747% CO^38,41,42^
Radiation	Xenon UV-lamp (spectral range 200–2200 nm) on a 13 mm sample spot (16 h light/8 h dark)^19,20,32^	Solar radiation (> 200 nm)^34,35^

Four samples of *X. parietina* were illuminated by the Xe-UV lamp in sample position (sp) 3, 4, 5, 6 and four samples were kept in darkness inside the experiment chamber, exposed to the atmospheric conditions in the sp 1, 2, 7, 8 (Fig. 2b). The Xe-UV lamp simulates the complete Martian solar spectrum with a spectral range from 200 to 2200 nm on a 13 mm diameter spot (Table 1). A comparison to the Sun’s spectrum on Mars can be seen in de Vera et al.^20^ and Schuerger et al.^33^. The UV lamp was active for 16 h and switched off for 8 h daily to simulate the mid-latitudes summer Sun’s diurnal cycle. The UV lamp turned on and off automatically. An Optometer X92 with an RCH-106-4 head was used to measure UV fluxes and doses at wavelengths ranging from 250 to 400 nm. The measured UV irradiance values for the irradiated sample positions were 15.8 W m^−2^ (sp3), 12.7 W m^−2^ (sp4), 14.2 W m^−2^ (sp5) and 14.0 W m^−2^ (sp6), with an average value of 14.2 W m^−2^ comparable to those elaborated by Cockell et al.^34^, ca. 17 W m^−2^, and Gómez-Elvira et al.^35^, 7–8 W m^−2^ for Mars’ surface depending on the latitude. Additionally, shorter simulations were performed as pilot studies for the 30-days exposure.

### Experimental design

Twelve samples (1 cm^2^ area each) have been prepared from the collected and reactivated lichen thalli for the pre-exposure analyses phase. The pre-exposure analyses consisted in Raman spectroscopy and Chlorophyll *a* fluorescence analysis. After 24 h, the pre-exposure photoefficiency was measured with an Imaging PAM (Walz GmbH, Germany). Four samples were then exposed to the Mars simulated atmosphere and irradiated by the Xe-UV lamp (spectral range 200–2200 nm, 16 h light/8 h dark) (FM, Full Mars samples positioned on sp3, sp4, sp5 and sp6), four samples were exposed to the Mars simulated atmosphere and kept in darkness (DM, Dark Mars samples positioned on sp1, sp2, sp7 and sp8) and four samples were kept in the growth chamber at 25 °C, with 12 h dark and 12 h light at 50 μmol m^−2^ s^−1^ PAR photons, daily wetted as external controls^28,31^. During the simulation, the photosynthetic activity of the FM and DM samples was measured every hour with a Mini PAM, thanks to the experiment chamber’s sample carousel (Fig. 2b). Simultaneously, thermophysical conditions inside the experiment chamber were monitored with a Labview program. The simulation lasted 30 days. After exposure the samples were not rehydrated immediately but were frozen for the next day’s post-exposure analyses, in order to avoid possible lichen re-activation. Small segments from the samples (FM, DM and external controls) were cut for the Raman spectroscopy as previously done for the pre-exposure phase. Further segments were retrieved from the FM, DM samples and external controls and stored in a freezer at -18 °C for transmission electron microscopy, carried out later at the Biomorphology Laboratory at the University of Florence, Italy. The post-exposure analyses consisted of the Raman spectroscopy, after which the FM and DM samples were rehydrated for the recovery phase. During this last phase, the FM, DM and external control samples were kept in the growth chamber at 25 °C, with 12 h dark and 12 h light at 50 μmol m^−2^ s^−1^ PAR photons, wetted daily for 8 days^28,31^. Every day of the recovery phase—with exception of the weekend—the FM, DM and external control samples were measured with the Imaging PAM to assess photosynthetic activity and verify their recovery over time. Overall, the photosynthetic efficiency measurements—carried out at different times were: before the treatment (pre_exp), after the treatment (post_exp) and 24 h (1 day), 48 h (2 days), 72 h (3 days), 96 h (4 days), 168 h (7 days) and 192 h (8 days) after the beginning of the recovery phase.

### Chlorophyll a fluorescence analyses

Photosynthetic activity of the lichen photobiont was inferred from the Chl *a* fluorescence as an indicator of the photosystem II (PSII) efficiency^13,19,20,28^. The Chl *a* fluorescence was measured during the 30-days simulation every hour through a Mini PAM instrument (Mini PAM model II interfaced with the PASLAB facility) (Fig. 2a). The Mini PAM light fiber was ~ 1.5 cm distant from the sample. Fluorescence measurements were expressed as PSII quantum yield (*Y*), through the following equations: (i) for light-adapted lichens as Y(II) = (F_M_′ − F)/F_M_′^43^ and (ii) for dark adapted lichens as F_V_/F_M_ = (F_M − _F_0_)/F_M_^44^, where F (light-adapted lichens) and F_V_ (dark-adapted lichens) are the natural fluorescence values of the samples measured briefly before the Mini PAM saturation pulse triggering^20^. F_M_ is the maximum fluorescence measured after dark adaptation and F_0_ is the minimum fluorescence yield^44^. F_M_′ is the maximum fluorescence reached during the saturation pulse, measured in light conditions when all PSII reaction centers are open. The effective PSII quantum yield *Y*(*II*) is obtained from the ratio of fluorescence intensities as described by (i). Instead, the maximal PSII quantum yield F_V_/F_M_ is obtained as described by (ii). Any inhomogeneities of fluorescence excitation intensity can be interpreted in terms of differences in photosynthetic activity^45^. The Mini PAM, according to light/dark conditions, calculated the effective rate of photochemical energy conversion, displayed on the Mini PAM as the *yield* value. Samples were exposed to a flashing light for 1 s with an excitation pulse of red light (650 nm).

An Imaging PAM was used to assess the photobiont photoefficiency before, after and in the next 8 days after the exposure. The Imaging PAM was equipped with a Mini Measurements Head connected to an IMAG-K5 camera (aperture f.2, 640 × 480 pixels, 96 dpi). The Mini Head is equipped with a LED-array platform featuring twelve high-power LED-lamps each furnished of collimating optics, which were arranged in four groups of three LEDs. The red-light LEDs (IMAG-MIN/R 650 nm, with an emission peak at 620 nm) provided the saturation pulse excitation light. The LED-array platform was mounted at the working distance of 7 cm above the samples. The Imaging PAM retrieved samples’ imaging through F_V_/F_M_ fluorescence average values of the selected area on the sample. Fluorescence measurements were performed in dark-adapted conditions. Lichen samples were hydrated and dark-adapted (covering the Petri dish with a black cloth) for 10 min before Imaging PAM measurements^28,31^.

### Raman spectroscopy

Raman measurements were performed with a Confocal Raman Microscope WITec Alpha300 Raman System (Oxford Instruments, UK) at room temperature and ambient atmospheric conditions. The Raman laser excitation wavelength was 532 nm, a spectral resolution of 4–5 cm^−1^ and 600 l/mm grating. A Nikon 10 × objective with a 0.25 numerical aperture was used to focus the laser on a 1.5 μm spot. The surface laser power was set at 1 mW. A spectral calibration was performed with a pure silicon test sample^46^. In order to avoid eventual sample damaging by Raman spectroscopy, a small segment from each of the samples was cut on purpose. For each segment (4 Full Mars and 4 Dark Mars, before/after the exposure) 4 spots were identified. On each spot, a line scan (transect) was retrieved with 1 s acquisition time for 1 × accumulation on 50 points (50 spectra), obtaining 200 spectra per sample. The acquisition setting was chosen in order to avoid signal saturation from photosynthetic pigments’ fluorescence, specifically chlorophyll. In fact, Raman measurements were performed in order to identify changes in carotenoid peaks’ features. *β*-carotene and xanthophylls are the main molecules responsible for the carotenoids’ Raman signal and the typical Raman signal expected consisted of three main carotenoid peaks^46^. Two strong peaks at 1515 cm^−1^ and 1150 cm^−1^, characteristic of in-phase C = C (ν_1_) and C–C stretching (ν_2_) vibrations of the polyene chain in carotenoids. Additionally, in-plane rocking modes of CH_3_, groups attached to the polyene chain coupled with C–C bonds occurred in the 1000 cm^−1^ region^46,47^.

We proceeded by evaluating (i) the Signal to Noise Ratio (SNR) values of the retrieved spectra, and (ii) the difference before/after the exposure of the carotenoid’s peaks features to compare the effects of the Mars-like conditions on the exposed samples. The SNR for carotenoids peaks in the spectra was defined as the height (Amp) of the 1515 cm^−1^ peak divided by the noise represented as the standard deviation of the spectral region near the Raman peaks (700–900 cm^−1^)^46^. The accumulated spectra were divided into three different classes^47^. Spectra of class 1 show a strong SNR with the three characteristic peaks dominating the spectra (SNR > 20). Class 2 spectra have a medium/low SNR with the 1000 cm^−1^ peak fading (5 < SNR < 20). Class 3 spectra are classified by null/non-detectable peaks (SNR < 5)^47^. The peaks’ features of FM samples spectra and DM samples spectra—the height or amplitude (Amp), the width at half height (w) and the peak position on the wavenumber (x)—were retrieved from the class 1 spectra (SNR > 20) by fitting the resulting averaged spectrum by using a Lorentz function^46^. Lorentzian fit was performed on the Project FIVE 5.0 software (WITec Suite FIVE).

### Transmission electron microscopy

Transmission electron microscopy was carried out at the Biomorphology Laboratory of the University of Florence, Biology Department. After the exposure, small segments were cut from the exposed samples and then were sealed in Eppendorf tubes and stored in freezer at − 18 °C until microscopy analyses. Segments had to be fixed, dehydrated, resin-embedded and stained fo TEM observations^48^. Fixation and inclusion processes were used in order to prevent the decomposition of the segments and to preserve their chemical and physical properties. The segments were fixed in 2.5% glutaraldehyde (C_5_H_8_O_4_) in 0.1 M of phosphate buffer (pH 7.2) for 4 h. Then, they were subjected to a 12 h washing in 0.1 M of phosphate buffer (pH 7.2). The third step involved fixation in 1% osmium tetroxide (Os_4_O) in the same buffer for 90 min and then, a 20 min washing in 0.1 M of phosphate buffer (pH 7.2)^49^. The phosphate buffer served to convey the fixative, to avoid modifications of pH, osmolarity and ionic compositions. Segments were then gradually dehydrated with ethanol to prepare them for inclusion. The resin’s embedding allowed to obtain a hard material that can be finely sectioned. After a last step in propylene oxide, the Spurr epoxy resin^50^ was gradually introduced into the segments. In the first step, they were pre-included in propylene oxide and Spurr in a 1:1 proportion for 1 h. Then, the quantity of resin was doubled up to a proportion of solvent and Spurr of 1:2 for 1 h. In the third step, segments were treated with pure resin in an oven at 45 °C for 1 h and in the end, included in the HistoMold embedding box at 70 °C in the stove for 12 h. The included segments were cut with an Ultramicrotome Reichert Jung Ultracut E (Leica Biosystems GmbH, Germany), obtaining sections with a thickness between 50 and 100 nm using diamond and glass blades. The sections were placed on the surface of a container with distilled water and then transferred to a copper grid for TEM observations. Some sections were put on a slide and stained with 0.1% Toluidine Blue and 0.1% diluted sodium carbonate in H_2_O, then observed under the LEICA LEITZ DM-RB FLUO optical transmitter light microscope (Leica Biosystems GmbH, Germany). TEM observations were done with a PHILIPS EM201 transmission electron microscope (Philips, Netherlands)^48^.

### Statistical methods

For both the Chl *a* fluorescence analyses (Mini PAM, during the exposure and Imaging PAM, before/after the exposure plus recovery period), data were analyzed fitting linear mixed-effects models (LMMs) in a repeated measurements ANOVA design, using sample identity as a random factor to account for the temporal correlation of observations. Time was used as an ordinal variable because the relationship between *Y* and time was not a simple linear regression. *Y* was used as response variable and the two treatment conditions (FM and DM) and time of recovery as explanatory variables in a full factorial design. ANOVA type II Wald chi-square test method was used to verify significance of the fixed effects and of associated interaction factors. Data normality was checked with the Shapiro–Wilk test. In particular for Mini PAM analyses, the Pearson’s correlation coefficient test was performed to verify linear correlation between *Y* and the thermophysical parameters of the experiment chamber (T, P, hum and tau/frost-point).

Raman data were elaborated recovering the carotenoids peaks’ features values (Amp, w and x) from the SNR > 20 spectra (see “Raman spectroscopy” paragraph). Specific dataset consisting of the before/after feature’s values were separated by treatment. Each dataset was analyzed with one-way ANOVA to verify eventual significant difference between before and after the exposure features’ values. Welch Two Sample t-test was performed on the datasets too for one-way ANOVA proofing. The non-parametric Kruskal–Wallis test was performed—instead of ANOVA—when carotenoids peaks’ feature values were not distributed normally.

All the analyses were run with the open-source software RStudio v. 1.4.1106. ANOVA computations were performed using the *Anova* function of the *car* package version 3.0-10 for ANOVA type II Wald chi-square test and the *aov* function (in base R) for one-way ANOVA test. LMM computations were performed using the *lmer* function of the *lme4* package version 1.1-12 for fitting the models.

## Results

### Chlorophyll a fluorescence analysis

**Figure 4:**
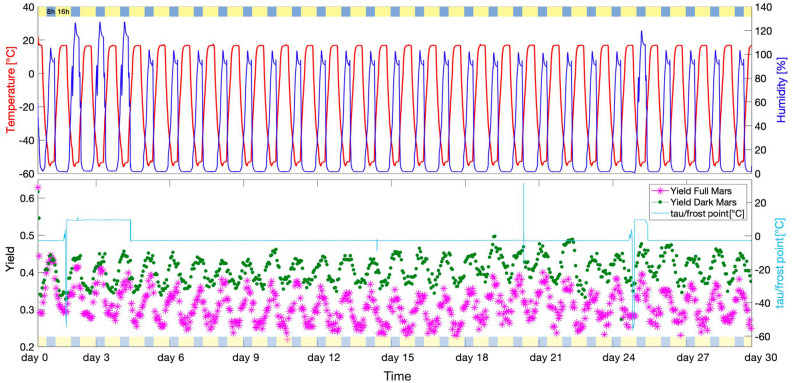
Above, temperature (red line) and humidity (blue line) day-night cycles as performed in the PASLAB at DLR Berlin. The light-blue/yellow stripe represents the day and night photoperiod related to the UV lamp on/off. Below, the cyan line represents the gas mixture humidity frost-point. The magenta stars represent the Yield values of Full Mars FM samples and the green dots represent the Yield values of Dark Mars DM samples. See Table S1 for ANOVA results.

The results of the *in-situ* photo-efficiency analysis is shown in Fig. 4 with the corresponding Mars-like conditions in the top section of the image. The measured UV irradiance values for the irradiated samples were 15.8 W m^−2^ (sp3), 12.7 W m^−2^ (sp4), 14.2 W m^−2^ (sp5) and 14.0 W m^−2^ (sp6), with an average value of 14.2 W m^−2^. The final UV-radiation cumulative absorbed dose was 24.5 MJ m^−2^. In Fig. 4, after the first 4 h, a drop in FM and DM yield *Y* values, of 54% and 42%, respectively, was observed. After that, the photosynthetic activity did not remain constant during the experiment, following a cyclic “up and down” trend. Specifically, the Mini PAM measured the highest *Y* values at night (8 h of darkness), corresponding to maximum relative humidity and a temperature of − 55 °C, and the lowest *Y* values during day (16 h of light for FM samples), corresponding to minimum relative humidity and a temperature of 16 °C. The data in Table S1 confirms the significant difference between high and low peak *Y* values during the experiment and the significant difference between the two treatments (FM and DM) (*p* < 0.001). Pearson’s correlation test indicated significant correlation (*p* < 0.001) between *Y* values and temperature (R = − 0.62 FM, R = − 0.52 DM) and humidity (R = 0.59 FM, R = 0.44 DM) (Fig. S1). *Y* values did not show highly significant correlation with pressure (R = 0.23 *p* < 0.001 FM, R = 0.12* p* < 0.05 DM) and tau/frost-point (R = 0.14 *p* < 0.05 FM, R = 0.065 *p* = 0.1 DM) (Fig. S1). As reported in Fig. 4, between the 2nd and 4th day and the 25th night, the frost point temperature reached ~ 9.0 ± 0.2 °C because the CO_2_ gas bottle was empty and therefore had to be changed. Since only the dry air gas flow of 0.5 l h^−1^ as inserted in those time-periods, humidity values showed an increase because the vacuum pump was not extracting the experiment chamber atmosphere (because of the lower inlet air gas flow).

**Figure 5:**
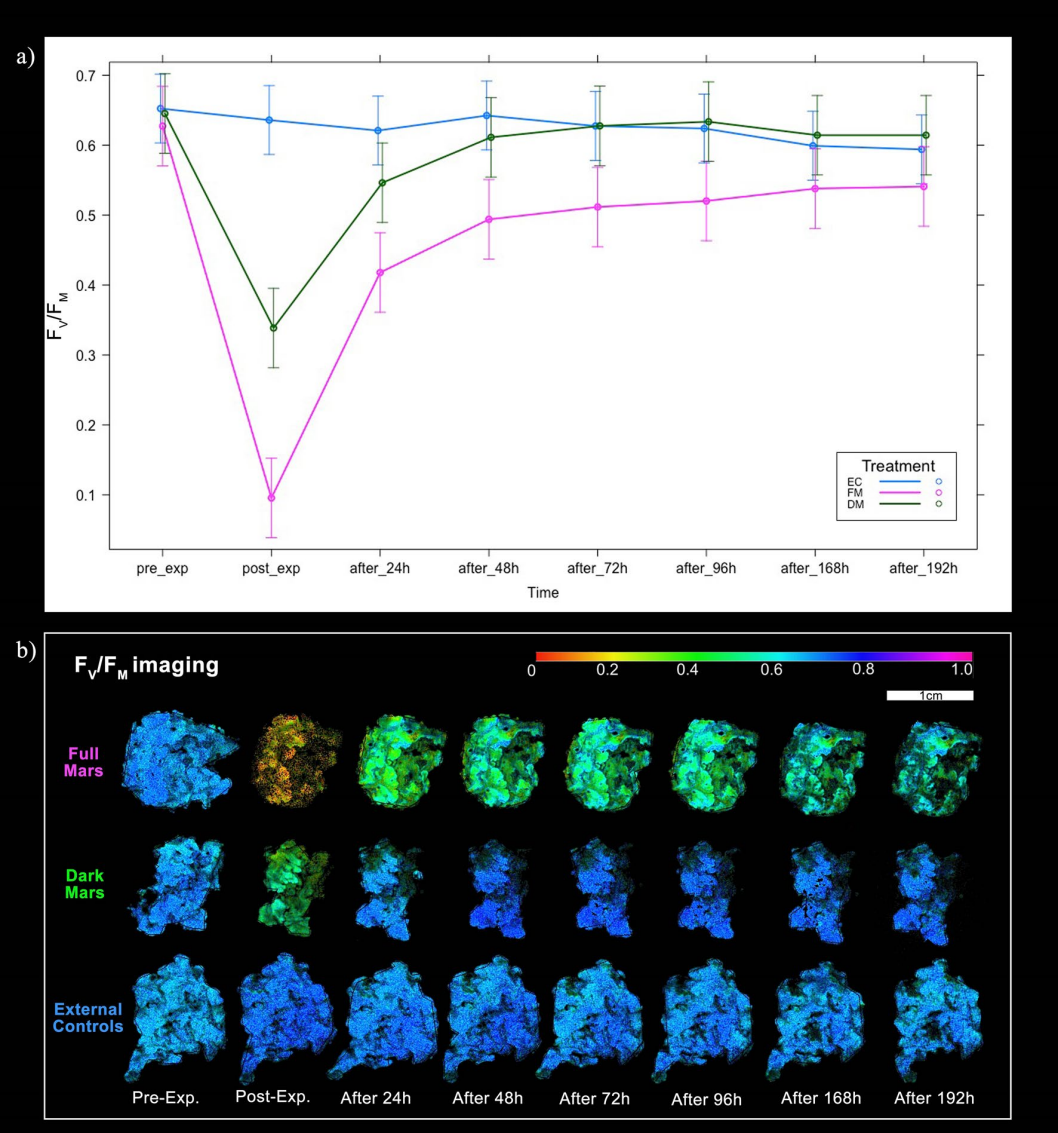
(**a**) Variation of the PSII (Y = F_V_/F_M_) efficiency before (pre_exp), after (post_exp) and 24 h, 48 h, 72 h, 96 h, 168 h and 192 h after the treatment. Blue line = external control, EC; magenta line = full Mars, FM; green line = dark Mars, DM. Error bars stands for confidence intervals. See Table S3 for ANOVA results. (**b**) Fluorescence (F_V_/F_M_) imaging of three samples’ treatments (Full Mars, Dark Mars and External Controls) before (pre-exp.), after (post-exp.) and 24 h, 48 h, 72 h, 96 h, 168 h and 192 h after the treatment performed with Imaging PAM instrument. After simulation, small segments for TEM observations and Raman spectroscopy were cut from Full Mars and Dark Mars samples. Lichen material size and color bar values’ legend is reported on the bottom right of the image.

The results of the recovery photoefficiency analyses are shown in the Fig. 5a and Table S2. The maximum quantum yield of primary photochemistry (Y = F_V_/F_M_) changed significantly after the treatment in both the experimental conditions (Table S3, *p* < 0.001). Yield (F_V_/F_M_*)* values of the FM and DM samples showed a significant decrease compared to pre-exposure values. DM values reached the drop-down fall value of 0.339 ± 0.083 (Mean ± SD). Instead, FM values reached a lower value of 0.096 ± 0.042, crossing the threshold of 0.200, which is considered the photoinhibition limit^51^. Once the recovery period began, F_V_/F_M_ values increased significantly depending on the treatments. In 24 h, FM values recovered already 0.418 ± 0.023 and DM values reached 0.546 ± 0.122. For both the treatments, samples showed similar recovery trends, with DM values reaching the maximum at 0.634 ± 0.051 (after_96h). Nevertheless, after the 72 h of recovery, FM values did not increase more than 0.541 ± 0.007 (after_192h). Consequently, FM samples were not able to recover the initial F_V_/F_M_ values. The recovery conditions were the same of the control samples (see “Materials and Methods” section). Control samples’ F_V_/F_M_ values ranged between 0.653 ± 0.020 (pre_exp) and 0.594 ± 0.040 (after_192h). During weekends, the samples in the growth chamber were not hydrated (even if on Fridays, thalli were consistently wetted) and probably for this reason it was observed a decrease of F_V_/F_M_ values of control samples at the end of the recovery period. F_V_/F_M_ imaging, reported in Fig. 5b, shows the FM sample’s photoefficiency depression of the thallus after treatment (yellow-brownish false colors), and after reactivation, the false colors shifting from brown to green and dark-green, never reaching again blue, over the next 192 h^52^. On the contrary, DM samples’ F_V_/F_M_ imaging shows a green/dark green false color for post-exp. imaging, recovering after that the blue false color.

The minimum fluorescence yield F_0_ values are reported in the Fig. S2 and Table S4. As reported in Table S5, there is no significant difference between treatments. After exposure, FM and DM F_0_ values reached 0.136 ± 0.054 and 0.124 ± 0.010, respectively. For both the treatments’ samples showed a decreasing trend over the recovery period with DM samples being quicker in the drop fall (Fig. S2 and Table S4). At the end of the recovery period, FM and DM F_0_ values were 0.081 ± 0.024 and 0.083 ± 0.038, respectively. Control samples’ F_0_ values ranged between 0.122 ± 0.031 (after_192h) and 0.151 ± 0.021 (after_24h) over the recovery period.

Additionally, we performed shorter simulations of different durations. The 3-h simulation aimed to intercept the Chl *a* fluorescence (Yield) decreasing curve at the application of the Mars-like conditions to evaluate how rapidly the photoefficiency decreases. The 7-days simulation was performed in order to assess eventual changes in photoefficiency recovery with the 30-days simulation samples. The shorter simulation results are reported in Supplementary Materials.

### Raman spectroscopy

**Figure 6:**
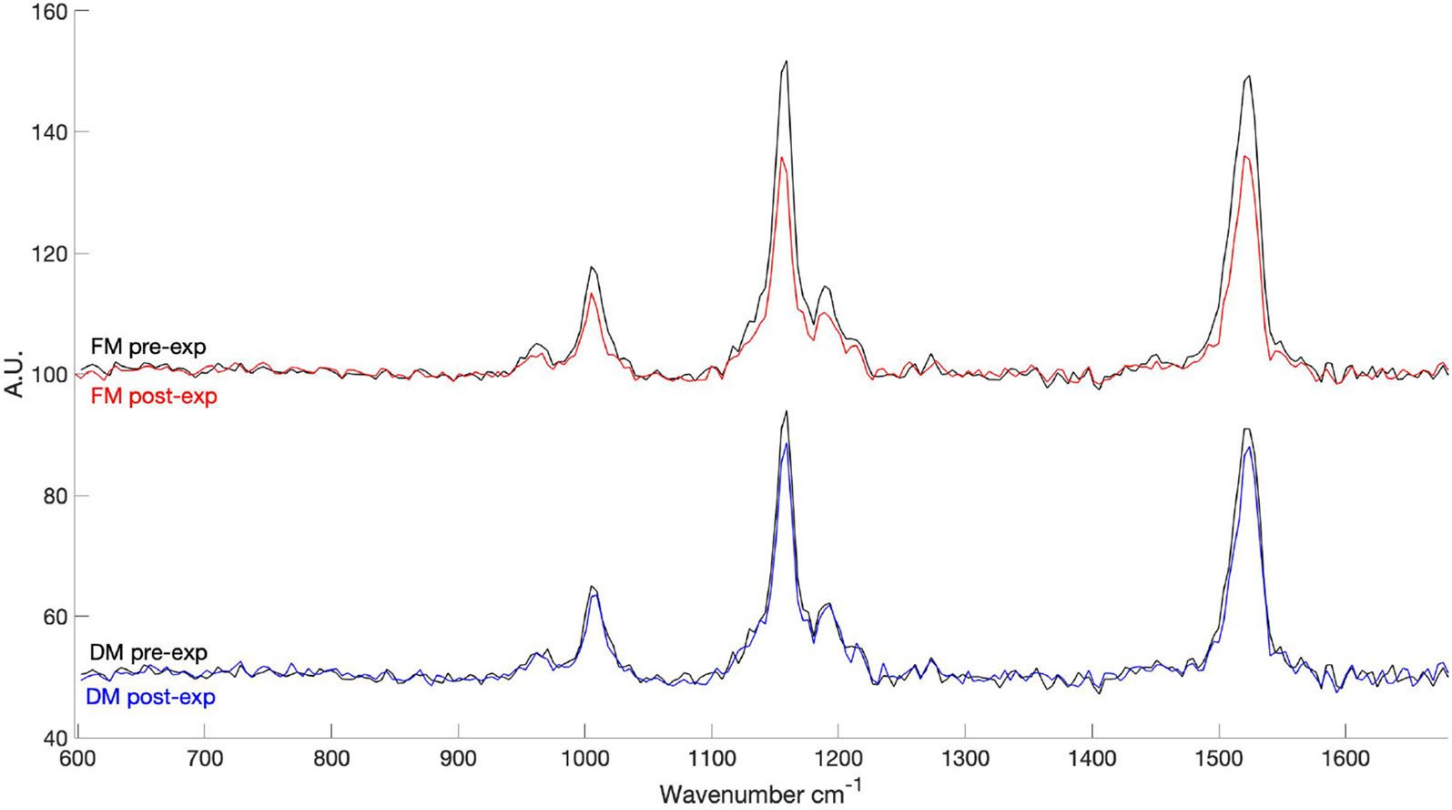
Above comparison between mean FM pre-exp. Raman spectra (black line) and mean FM post-exp. Raman spectra (red line). Below) comparison between mean DM pre-exp. Raman spectra (black line) and mean DM post-exp. Raman spectra (blue line).

Figure 6 shows the comparison between the before/after FM and DM Raman spectra. A typical carotenoid spectrum, with the three peaks at 1000, 1150, and 1515 cm^−1^, was successfully measured for all samples but with a clear decrease in peaks’ intensities after exposure. To quantitatively compare the spectra according to the treatment, we considered the peak’s feature Amp (amplitude, height or peak intensity), w (FWHM, full width at half maximum) and x (peak position on the wavenumber) of spectra with SNR > 20 (see “Materials and Methods” section). A non-parametric Kruskal–Wallis test was performed for all the FM carotenoids peaks’ position values (x) and for the DM 1515 cm^−1^ position values (x) because data were not normally distributed. The average carotenoid peaks’ features (Amp, w and x)—before and after the exposure—are reported in Tables S6 and S7. Figure S3 shows the 1000 cm^−1^ peak’s features compared before/after the exposure for both the treatments FM (on the left) and DM (on the right). In FM samples, Amp, w and x differences were significant (*p* < 0.001, Tables S8 and S9). In DM samples, w and x differences were both significant (*p* < 0.001, Tables S8 and S9), but Amp difference was not (Table S8). Fig. S4 shows the 1150 cm^−1^ peak’s features (Amp, w and x) compared before/after the exposure for both the treatments FM (on the left) and DM (on the right). In FM samples, Amp and x differences were significant (*p* < 0.001, Table S8 and Table S9), but w difference was not highly significant (*p* < 0.05, Table S8). In DM samples, Amp (*p* < 0.1), w (*p* < 0.05) and x (*p* < 0.05) differences were not highly significant (Tables S8 and S9). Fig. S5 shows the 1515 cm^−1^ peak features (Amp, w and x) compared before/after the exposure for both the treatments FM and DM. In FM samples, Amp, w and x differences were significant (*p* < 0.001, Tables S8 and S9). In DM samples, Amp difference was not highly significant (*p* < 0.1, Table S8), w difference was significant (*p* < 0.001, Table S8) and x difference was not significant (Table S9).

### Transmission electron microscopy

#### Characterization of external control samples

**Figure 7:**
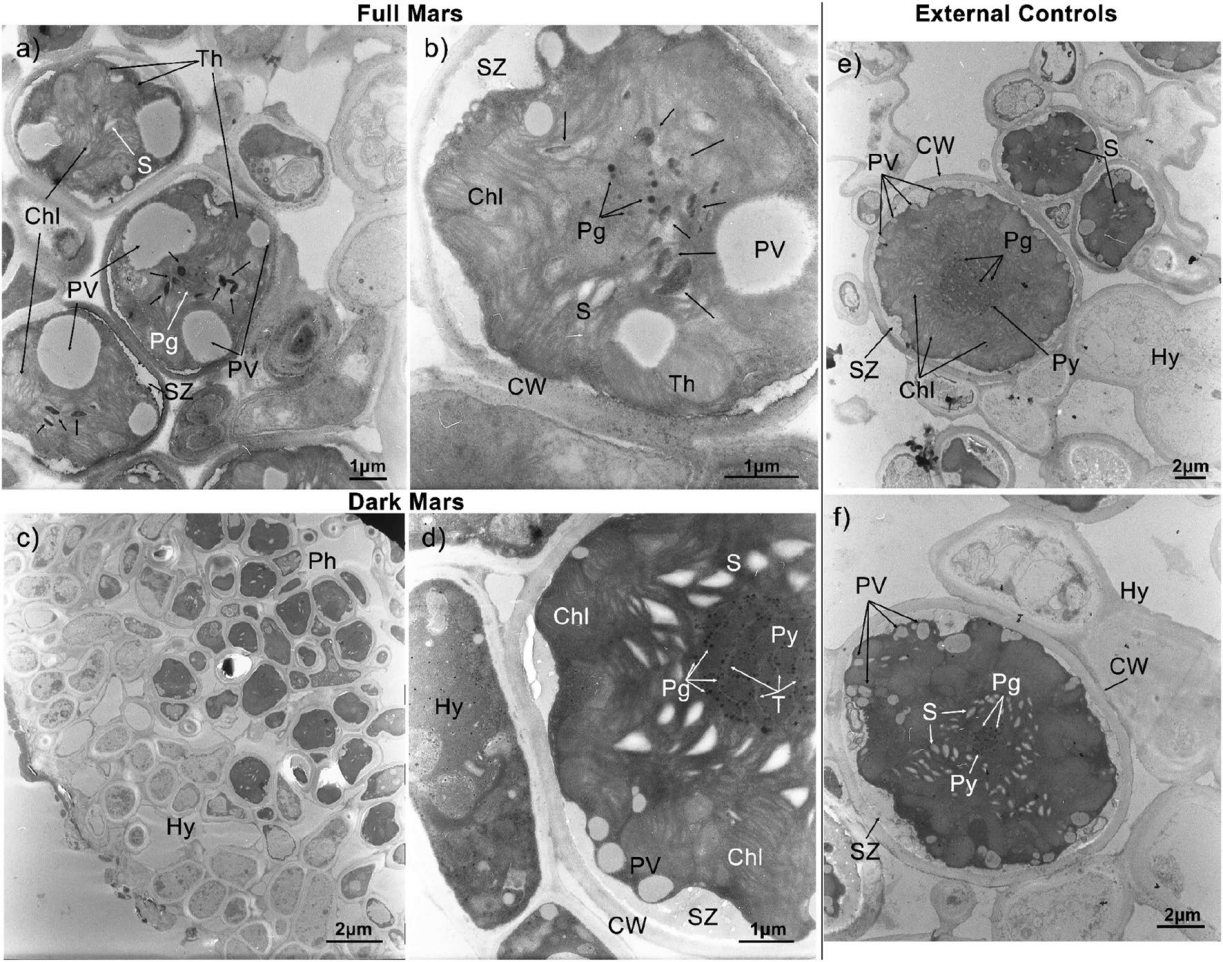
TEM ultrastructural analysis of photobiont *Trebouxia sp*.; (**a**, **b**): Full Mars (FM); (**c**, **d**): Dark Mars (DM) and (**e**, **f**): External Controls (EC). Each image is reported with a reference size bar: (**a**) 1 µm; (**b**) 1 µm; (**c**) 2 µm; (**d**) 1 µm; (**e**) 2 µm; (**f**) 2 µm. Abbreviation symbol stand for Chl, chloroplast; CW, cell wall; Hy, hyphae; Pg, pyrenoglobuli; Ph, photobionts; PV, peripheral vesicles; Py, pyrenoid; S, starch granules; SZ, secretion zone; T, tubules and Th, thylakoid. Black arrows indicate electron-dense lipid droplets.

The External Control samples’ ultrastructural characterization was based on the works of Hinojosa-Vidal et al.^53^, Meyer et al.^54^ and Molins et al.^55^. Figure 7 shows two images (Fig. 7e,f) of the external control sample. In Fig. 7e (2 µm size bar), there is the photobiont cell with visible thylakoid membranes (Chl)—forming the typical star-like chloroplast of *Trebouxia sp*.—and recognizable pyrenoid (Py) with pyrenoglobuli (Pg). Cell wall (CW), peripheral vesicles (PV) and secretion zone (SZ) are indicated too. The two small photobionts’ cells in the upper part of the image contain starch granules (S). The three photobionts are surrounded by hyphae (Hy). In Fig. 7f (2 µm size bar), an algal cell shows a high content of starch granules (S). These latter are arranged in a “star-like” pattern as the thylakoid membranes, that are not visible in the image. In the center of the cell, a pyrenoidal structure (Py) and pyrenoglobuli (Pg) are recognized.

#### Characterization of full mars and dark mars samples

Figure 7 shows two images (Fig. 7a,b) of the FM samples and two images (Fig. 7c,d) of the DM samples after the 30-days simulation. Figure 7a (1 µm size bar) shows a general disorganization in thylakoid membranes (Th), while chloroplasts (Chl) do not show the typical “star-like” shape anymore. The algal cells show a lower number of starch granules (S), few and bigger peripheral vesicles (PV) (as observed also by Molins et al.^55^) and fewer pyrenoglobuli in middle (Pg). Black arrows indicate supposed electron-dense lipid droplets^8,56–58^. Figure 7b (1 µm size bar) shows clearly the disorganization of the chloroplast (Chl) and related thylakoids’ membranes (Th), fewer pyrenoglobuli (Pg) in the middle, fewer starch granules (S) and bigger peripheral vesicles (PV). Black arrows indicate electron-dense lipid droplets. Figure 7c (2 µm size bar) reports the upper cortex layer, with the hyphal section (Hy), and the gonidial layer with photobionts (Ph), which seems to be compressed. Figure 7d (1 µm size bar) shows a photobiont cell surrounded by hyphae (Hy). In DM samples, the photobiont’s ultrastructure is more similar to the control samples than in FM samples. The “star-like” chloroplast (Chl), with the starch granules (S) following the thylakoids’ membranes shape, and the pyrenoid (Py) in the middle with the pyrenoglobuli (Pg) linked to each other through the white tubules (T), are clearly distinguishable. Pyrenoids appear less dense in pyrenoglobuli content than the control samples.

## Discussion and conclusions

The lichen species *Xanthoria parietina* was able to survive the Mars-like conditions simulated at PASLAB for 30 days. The nature of symbiosis metabolic relationship, poikilohydry and anhydrobiosis features may have contributed to the survivability of the lichen in Mars-like conditions. The two simulated treatments (FM and DM) affected differently the lichen samples, showing remarkable differences in the photosynthetic parameters, both in situ and during the recovery. In the first hours of the 30-days simulation, FM and DM samples showed a drop in Yield values, which the lichens overcame in less than ~ 12 h^20^. The exposure of FM samples to a dose of 24.5 MJ m^−2^ may have led to a decrease of the FM Yield maximum and Yield minimum of the cyclic fluctuation (compared to DM samples), reaching minimum values close to ~ 0.200, which is considered the photoinhibition limit^51^. The periodic fluctuation shows a strong correlation with temperature and humidity cycles (Fig. S1). Chl *a* fluorescence Yield values are strongly dependent on the lichen water content and consequently, on the water availability in the surrounding environment because of their poikilohydry^52,59–61^. Additionally, the activity of PSII is a prerequisite to the acquisition of energy by the photolysis of water^7^. Since relative humidity reached its maximum during night, Chl *a* fluorescence Yield (for both the treatments) reached its maximum at night too. The Yield’s cyclic fluctuation suggests higher photoefficiency at night—linked to the 100% of relative humidity—and almost photoinhibition during the day, especially for FM values when relative humidity was 0.1%. Since DM samples showed the same periodic fluctuation of FM samples, we exclude that the day-night fluctuation could have been related to the dark/light adaptation. The fluorimeter impulse let the reaction happen since photosynthesis depends on light, even during the simulation nighttime^20^. This means that the measurement of Yield value is due to light-dependent hydrolysis and electron replacement in chlorophyll molecules. Consequently, if humidity is available, the light reaction could work on Mars and should specifically work in dimmed sunlight time-periods (early morning and late evening), since—even on Earth—full sunlight inhibits optimal photosynthesis in algae and cyanobacteria^20^.

UV radiation plus vacuum/low pressure application proved to be more impacting on lichen photosynthetic performances^6,13,28^. The Yield optima values are not influenced directly by low pressure and high CO_2_ concentration^3^. Despite this, lichens express general high Yield values (almost always over ~ 0.200) when exposed to higher CO_2_ percentage levels maintained in low-pressurized Mars-like atmosphere (600 Pa). Therefore, high Yield values during the exposure may be related to low pressurized CO_2_ availability, and also to humidity diurnal cycles. Additionally, the high CO_2_ availability could represent an advantage for photosynthesis, suggesting the chance that the mycobiont constitutes a possible carbon source via respiration or via CO_2_ storage with the alga, delivering CO_2_ and up-taking O_2_ from photosynthesis^3^.

After the recovery time period, FM F_V_/F_M_ values did not reach the initial values, as previously investigated in Lorenz et al.^28^. On the other hand, DM F_V_/F_M_ post-exp. values decreased by 46%, recovering almost the 97% of the pre-exp. values in 48 h (Fig. 5a), suggesting null or less photoinhibition effects compared to FM samples. F_V_/F_M_ imaging (Fig. 5b) highlighted that the external lobes and specifically the lobes’ margins—representing the youngest parts of the thallus^62^—recovered first in both the treatments. F_0_ values—indicating the openness of PSII reaction centers in the dark^63^—show a decreasing trend over time during the recovery period for both the treatments with no significant difference (Fig. S2; Table S5). F_0_ is dependent on the chlorophyll content of the light-harvesting complex^64^ and consequently, on the structure of the antenna complexes^65^. In light of this, F_0_ trends after treatment (Fig. S2) may indicate damages related to the opening/closing mechanisms of the PSII reaction centers^28^, even if the treatments did not result significantly different (Table S5).

Furthermore, the time-length of the experiment may affect the F_V_/F_M_ post-exp. values and recovery speed with possible effects such as photoinhibition of PSII and delay in photosynthesis reactivation^66^. The reached F_V_/F_M_ values at the end of the recovery period suggest that PSII or chlorophyll structural damages may be similar in samples exposed to UV radiation for different time periods (see Supplementary Materials).

The hyphal matrix with the thallus structure and lichen substances may be involved in the lichen survivability. The secondary lichen substance parietin—deposited in the thallus’ upper cortex—is known for its blue-light, UVB and UVC screening properties^67^, occurring more efficiently in anhydrobiosis or desiccated state^27,67^ because of the better thermal dissipation mechanism of light energy^68^. Carotenoids also may have played a crucial role in the photobiont’s photoprotection. These substances fulfill two important functions, (i) the reaction centers’ photoprotection against high light intensities involving NPQ processes^69,70^ and (ii) reactive oxygen species (ROS) scavenging thanks to their anti-oxidant features^71–74^. The simulated conditions may have produced direct and/or indirect interaction-damages on photobionts cells^75^. These damages encompass photosystems, proteins and nucleic acids photodissociation/photoexcitation^76^ and/or ROS formation, involving lipid peroxidation, amino acids oxidation and enzymes’ activities alteration^66,77^. ROS quenching carotenoids (such as *β*-carotene) and xanthophylls, preventing ROS formation via xanthophyll cycle, may be consumed to prevent ROS harmful effects or photodegraded by UV radiation^76^. Additionally, carotenoids represent a crucial biomarker in astrobiology since their photoprotection-relatable features and high preservation under ionizing radiation when embedded in Martian soil simulants^46,47^.

Significant variations in FM sample carotenoids’ peak features were identified and DM samples did not show significant changes in carotenoids peaks’ Amp feature. Since Raman quantitative methods are frequently based on either band area or band height (Amp)^78–81^, we can assume that the Amp feature decrease may be related to the carotenoids’ consumption and to the reduction of carotenoids’ concentration in the FM samples. On the other hand, Raman band position (x) or width (w) are most often used for the analyte quantitative analyses related to composition, as crystallinity, stress or temperature^78,82^. Specifically, Raman band position depends on atomic masses and the strength of the chemical bond that joins them. Local molecular order, external forces, hydration, temperature and radiations can all affect the chemical bond’s strength^78,83,84^. Amp feature changes are mainly related to photodegradation processes triggered by UV radiation. On the other hand, w and x feature changes may be also related to atmospheric conditions such as temperature-humidity cycles^78,83,84^, since samples from both the treatments seem to be affected. Again, FM samples still show differences of higher significance pointing out a higher effect of photodegradation over the environmental conditions. These results agree with the Chl *a* fluorescence recovery analyses, where FM samples did not reach the beginning values over the recovery time. This suggests stronger and more harmful effects by the atmospheric conditions plus UV radiation and eventual damages to the photosystems, explaining the significant decrease of carotenoids’ peak heights. However, FM samples still resulted photosynthetically active in terms of Chl *a* fluorescence during the recovery time period, proving that lichens possess a complex photoprotective strategy to be successful in extreme environments^84^.

One of the key factors of lichens’ survivability is anhydrobiosis^66^, that produce visible and temporary alterations of the cellular ultrastructure^85,86^. Thus, photobiont cells are known to collapse temporarily upon dehydration under terrestrial conditions^85,86^. After rehydration, lichen photobionts recover their spherical shape^86^. On the contrary, permanent cellular collapses indicate desiccation-induced damage that may lead to death after several years of dehydration^86,87^. Vacuum desiccation is considered the main process affecting biological samples when exposed to space conditions while pressures similar to those on Mars produce less desiccation-related effects^86^. Our samples did not show any relevant shape alteration or protoplast’s collapse due to low pressure conditions exposure. DM samples’ photobionts did not exhibit significant changes, except for a decrease in the number of pyrenoglobuli related to dehydration/rehydration processes^88^. The function of these structures may be that of a reservoir of lipophilic pigments, used in case of necessity. The most significant differences were observed between control samples and FM samples. Since several peripheral vesicles were observed in control samples, we suppose that the large white bodies represent empty (because of the light color) vacuoles or enlarged peripheral vesicles possibly indicating cellular stress^8,55^. DM samples seem to preserve small and several peripheral vesicles. Several dark bodies were noticed only in FM samples. These electron-dense bodies could possibly represent lipid accumulations^56–58^ as a consequence of the lipids’ loss from the cellular and thylakoid membranes degradation and pyrenoid’s tubules disappearance^8^, indicating some degree of senescence^88^. Tubules’ collapse could be due to the water withdrawal from the lumen related to low pressures or to a change of the configuration of the dehydrated pyrenoid protein structure^89^. Photobionts exhibit general chloroplast disorganization in terms of thylakoid membranes—tending to swelling and curling—and pyrenoid matrix. In addition to low pressure and thermophysical Mars-like conditions, the lichen had to cope with UV radiation. In light of the Chl *a* fluorescence analyses and Raman results, we can confirm that UV radiation represented the most harmful factor^8,13^, agreeing with the ultrastructural analyses. Despite this, it appears that ultrastructural changes were reversible or, at least, that the cellular integrity was sufficiently high to maintain a proper photoefficiency and a functional lichen metabolism, as resulted from Chl *a* fluorescence analysis. Previous works showed that ultrastructural damage on cells of *R. geographicum* and *R. elegans* did not necessarily indicate a metabolic failure^13^. The interval 100–200 nm of solar UV radiation wavelength (vacuum-ultraviolet) is the most harmful part of the solar spectrum^8,86^ and since our samples were exposed to *λ* ≥ 200 nm light spectrum (representing Mars surface conditions), we may assume that they did not experience the most deleterious range of solar radiation.

Our findings showed for the first time that *X. parietina* is able to survive in conditions analogue to those found at the Martian equatorial belt for 30 days. However, further analyses need to be performed to investigate the lichen thallus’ interactions with Mars analogue soil and multiple exposures to assess photosynthesis adaptability to Mars conditions. Additionally, parietin’s photodegradation under UV radiation could be further investigated to understand its photo-preservation. Since light reaction could eventually occur on Mars in presence of water, further habitats—such as niches, that are dimmed/scattered light illuminated—may represent more suitable contexts for putative *X. parietina*-like organisms. Therefore, our results remarked that lichens are suitable organisms for experiments in astrobiology. The eco-physiological adaptability of different lichen species in extreme habitats is the key to understand their survival capacity, even in space and extraterrestrial environments. Moreover, the evaluation of lichens’ survivability in Mars-like conditions can also give us insights on the possible preservation of complex biomarkers on the surface of Mars, posing constrains to the surface exploration.

## Supplementary Information

Supplementary Information.

## Data Availability

Data is available under reasonable request to the corresponding author.
