# Supplementary material for: Survivability of the lichen Xanthoria parietina in simulated Martian environmental conditions
Source: Sci Rep. 2023 Mar 25;13:4893. doi: 10.1038/s41598-023-32008-6 (PMC10039903; doi:10.1038/s41598-023-32008-6)
Supplement: Supplementary file 1 — Supplementary Information. [file 41598_2023_32008_MOESM1_ESM.docx]

# **Supplementary Materials**

# **Survivability of the lichen *Xanthoria parietina* in simulated Martian environmental conditions**

## Christian Lorenz^1^, Elisabetta Bianchi^1^, Giovanni Poggiali^2,3^, Giulia Alemanno^4^, Renato Benesperi^1^, John Robert Brucato^3,*^, Stephen Garland^4^, Jörn Helbert^4^, Stefano Loppi^5^, Andreas Lorek^4^, Alessandro Maturilli^4^, Alessio Papini^1^, Jean-Pierre de Vera^6^ and Mickaël Baqué^4^

^1^Department of Biology, University of Florence, Via La Pira 4, 50121 Florence, Italy.

^2^LESIA-Observatoire de Paris, Université PSL, CNRS, Sorbonne Université, Université de Paris, 5 place Jules Janssen, 92190 Meudon, France.

^3^INAF-Astrophysical Observatory of Arcetri, Largo E. Fermi, 5, 50125 Florence, Italy.

^4^Planetary Laboratories Department, Institute of Planetary Research, German Aerospace Center (DLR), Ruthefordstraße 2, 12489 Berlin, Germany;

^5^Department of Environmental Sciences, University of Siena, Via P. A. Mattioli 4, 53100 Siena, Italy.

^6^Microgravity User Support Center (MUSC), Space Operations and Astronaut Training, German Aerospace Center (DLR), Linder Höhe, 51147 Cologne, Germany.

^*^email: john.brucato@inaf.it

# **Supplementary Information**

## Chlorophyll *a* fluorescence analysis

**3-hour simulation**

We performed simulations of different time length to assess changes in F_V_/F_M_ recovery trends. The aim of the 3-hour simulation was to intercept the Chl *a* fluorescence (Yield) decreasing curve at the application of the Mars-like conditions in order to evaluate how rapidly the photoefficiency decreases. For the experiment, four samples were positioned in the not-irradiated spots. UV radiation was excluded from this simulation and Chl *a* fluorescence was measured every five minutes. The results of the *in situ* photoefficiency analysis are shown in Fig. S6 with the correspondent applied thermo-physical conditions in the above section of the image. During the experiment, temperature decreased from 23 °C to 16 °C (red line), relative humidity decreased from 61% to 5% (blue line) and pressure decreased from 1020 hPa to 50 hPa (black line) (Fig. S6a). The gas mixture humidity frost-point stayed constant for the entire simulation. After 1h 31min circa, Chl *a* fluorescence values started to decrease when temperature reached 16 °C, humidity 25 % and pressure 400 hPa. From 1h 30 min to 2h 15min, every five minutes Yield values decreased of 13% on average. The decreasing trend stopped when temperature was 16 °C, humidity 11% and pressure 100 hPa. ANOVA type II Wald chi-square test confirms the significant difference between Yield values over the simulation time period (*p*<0.001). Fig. S6b shows the significant correlation between Yield values and the applied thermo-physical conditions’ parameters (T R=0.89 *p*<0.001, hum R=0.87 *p*<0.001 and P R=0.88 *p*<0.001).

**Comparison 7-day vs 30-day simulation recovery**

In the 7-day simulation, 12 samples (1 cm^2^ size each) were employed. Four samples in FM conditions (total absorbed dose 5.7 MJ m^-2^), four samples in DM conditions and four samples as external controls. Fig. S7 shows the recovery trends’ comparison between 30-days simulation’s treatments and 7-days simulation’s treatments for the maximum quantum yield of primary photochemistry (*Y* = F_V_/F_M_). Tab. S10 and Tab. S11 show the F_V_/F_M_ values and the significant difference between treatments in the 7-day simulation respectively. Fig. S7 and Tab. S12 show the significant difference between treatments from the two simulations (30-days vs 7-days). Specifically, the drop of F_V_/F_M_ values after the two simulation (sim.) time periods were highly different for the same treatment. The post-exposure values of FM samples for 30-day sim. and 7-day sim. were 0.096 ± 0.042 (comparable to the complete photoinhibition of the photosystems) and 0.331 ± 0.035 respectively. Instead, the post-exposure values of DM samples for 30-day sim. and 7-day sim. were 0.339 ± 0.083 and 0.537 ± 0.059, respectively. Despite this, the after_192h values were close among the two treatments. FM values in 30-day sim. and 7-day sim. were 0.541 ± 0.007 and 0.559 ± 0.030 respectively. DM values in 30-day sim. and 7-day sim. were 0.614 ± 0.058 and 0.643 ± 0.032 respectively. This may suggest that the simulation’s duration mainly affects the F_V_/F_M_ post-exp. values and recovery speed with possible photoinhibition of PSII and delay in photosynthesis reactivation. UV radiation exposure affects specifically the observed FM recovery trend that in both simulations reaches ~ 0.550 at the end of the recovery period. This last consideration may suggest that PSII or chlorophyll structural damages may be similar in FM samples of both the simulations, independently from the exposure’s duration.

#
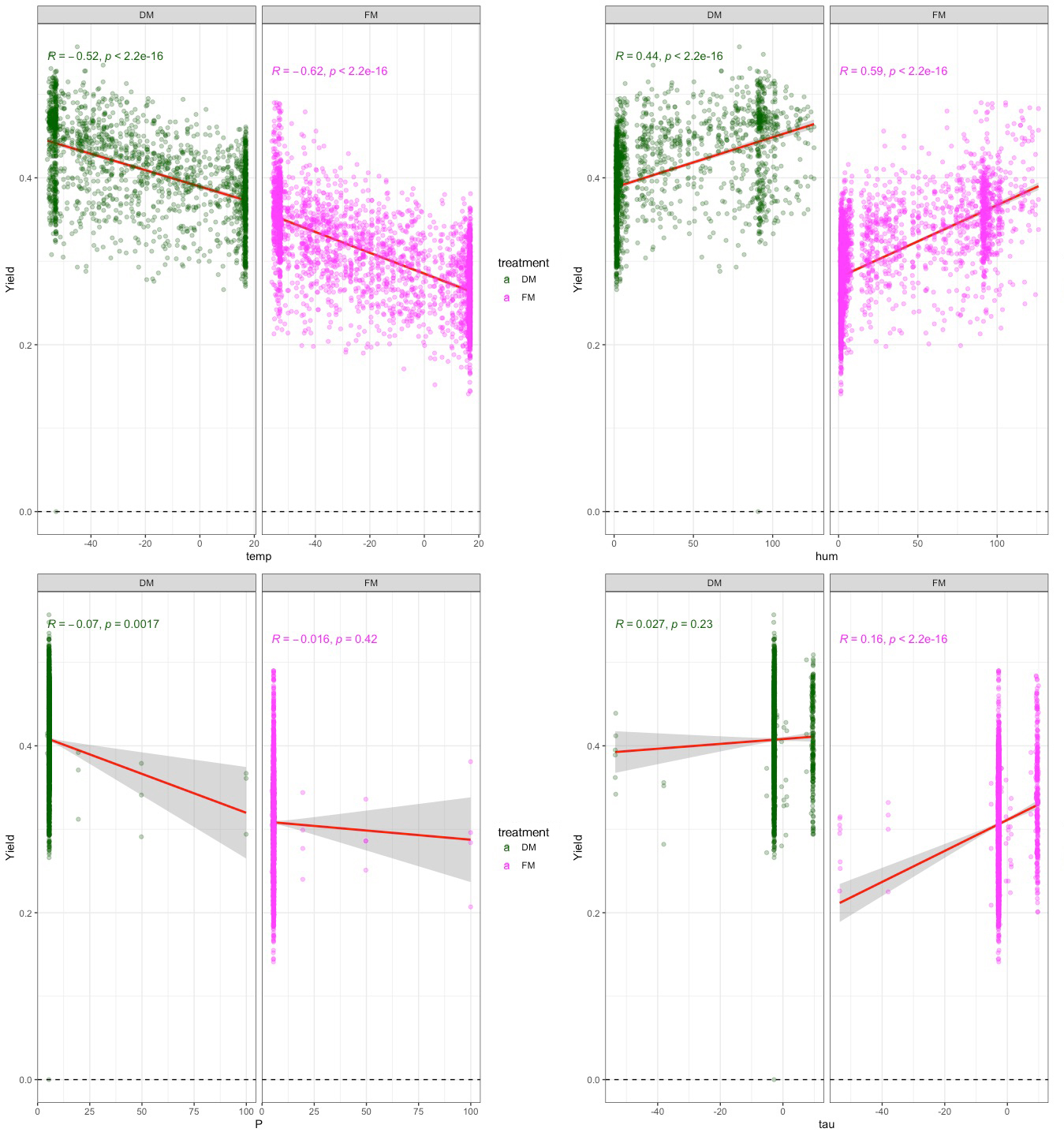
**Supplementary Figures**

Figure S1 - Above on the left) Pearson’s correlation test plot between Yield and temperature indicated a significant correlation in both the treatments (FM R=-0.62 p<0.001, DM R=-0.52 p<0.001). Above on the right) Pearson’s correlation test plot between Yield and humidity indicated a significant correlation in both the treatments (FM R=0.59 p<0.001, DM R=-0.44 p<0.001). Below on the left) Pearson’s correlation test plot between Yield and pressure did not indicate a significant correlation in both the treatments (FM R=-0.016 p=0.42, DM R=-0.07 p<0.05). Below on the right) Pearson’s correlation test plot between Yield and gas mixture humidity frost-point did not indicate a highly significant correlation in both the treatments (FM R=0.027 p=0.23, DM R=0.16 p<0.001).

Figure S2 - Variation F_0_ values before (pre_exp), after (post_exp) and 24 h, 48 h, 72 h, 96 h, 168 h and 192 h after the treatment. Blue line = external control, EC; magenta line = full Mars, FM; green line = dark Mars, DM. Error bars stands for confidence intervals. See Tab. S5 for ANOVA results.


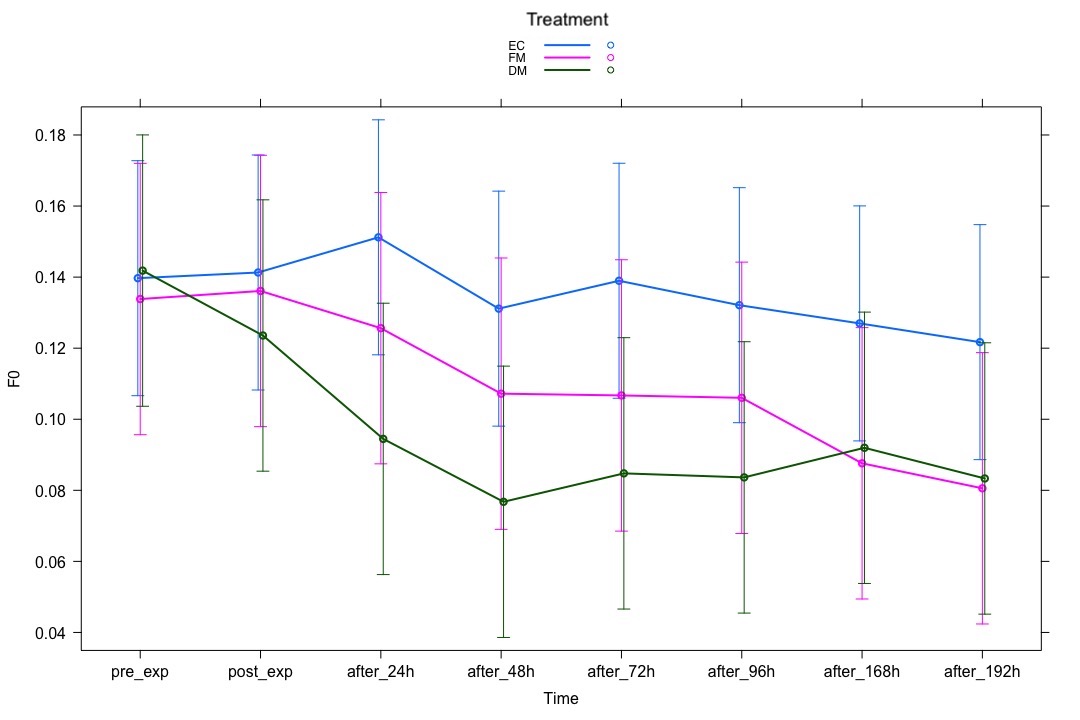


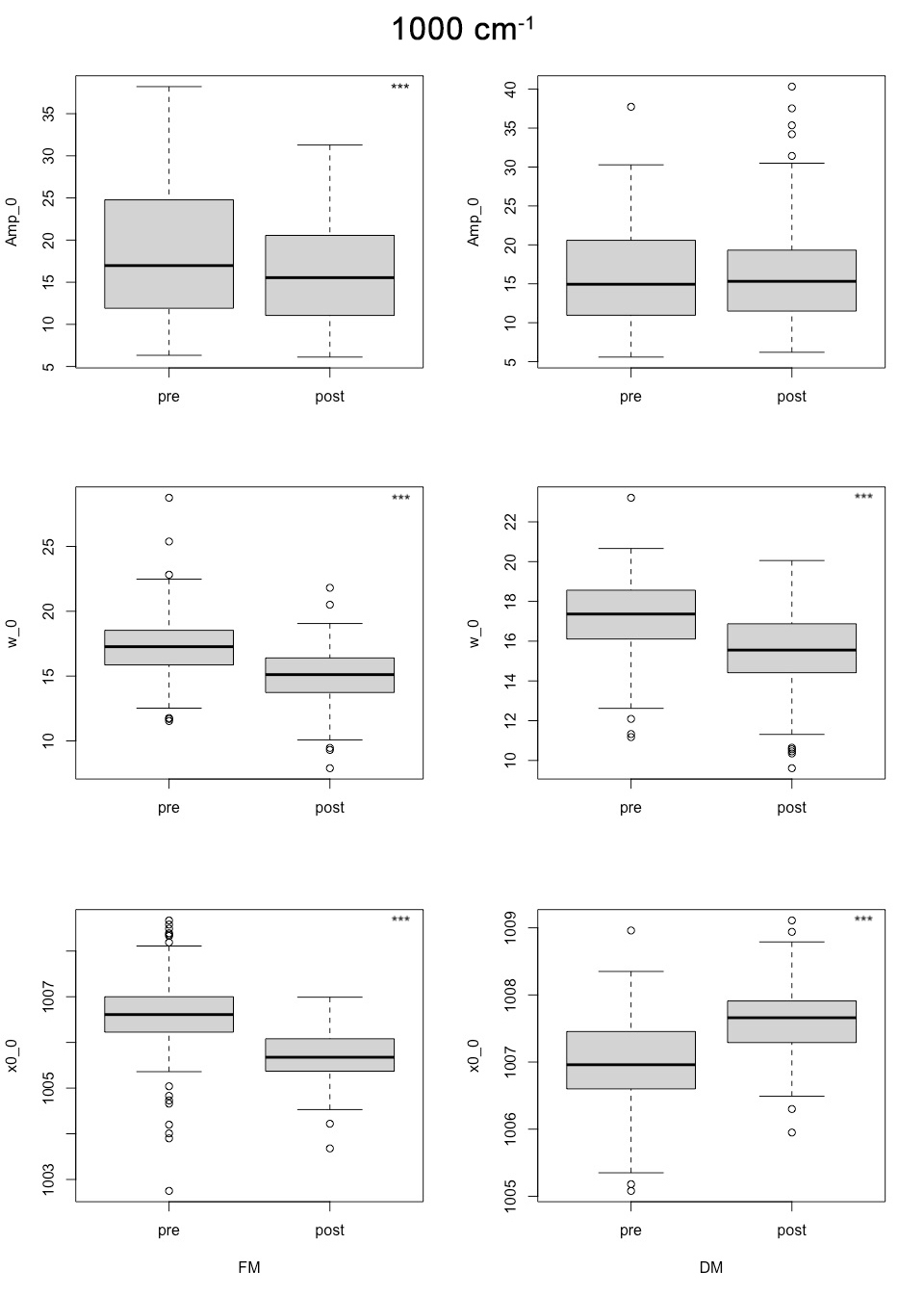
Figure S3 – 1,000 cm^-1^ peak’s features (Amp first row, w second row and x third row) boxplots representing differences between the pre-exp. and post-exp. values. On the left, FM values. On the right, DM values. One-way ANOVA test was performed for Amp and w (Tab. S8). Non-parametric Kruskal-Wallis test was performed for FM x (Tab. S9). Significance symbols are reported on the upper right corner of each boxplots.


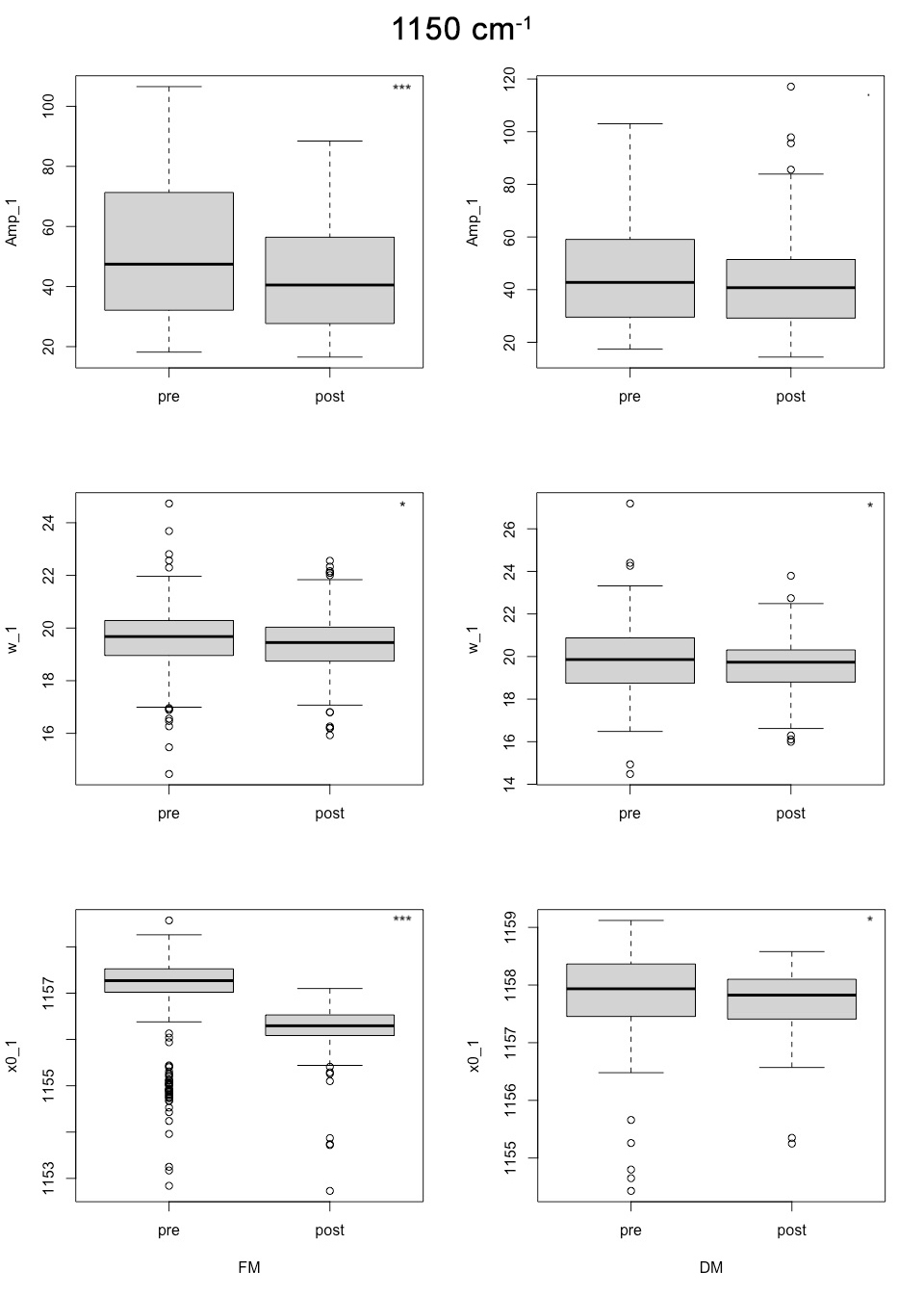
Figure S4 – 1,150 cm^-1^ peak’s features (Amp first row, w second row and x third row) boxplots representing differences between the pre-exp. and post-exp. values. On the left, FM values. On the right, DM values. One-way ANOVA test was performed for Amp and w (Tab. S8). Non-parametric Kruskal-Wallis test was performed for FM x (Tab. S9). Significance symbols are reported on the upper right corner of each boxplots.

Figure S5 – 1,515 cm^-1^peak’s features (Amp first row, w second row and x third row) boxplots representing differences between the pre-exp. and post-exp. values. On the left, FM values. On the right, DM values. One-way ANOVA test was performed for Amp and w (Tab. S8). Non-parametric Kruskal-Wallis test was performed for FM and DM x (Tab. S9). Significance symbols are reported on the upper right corner of each boxplots.


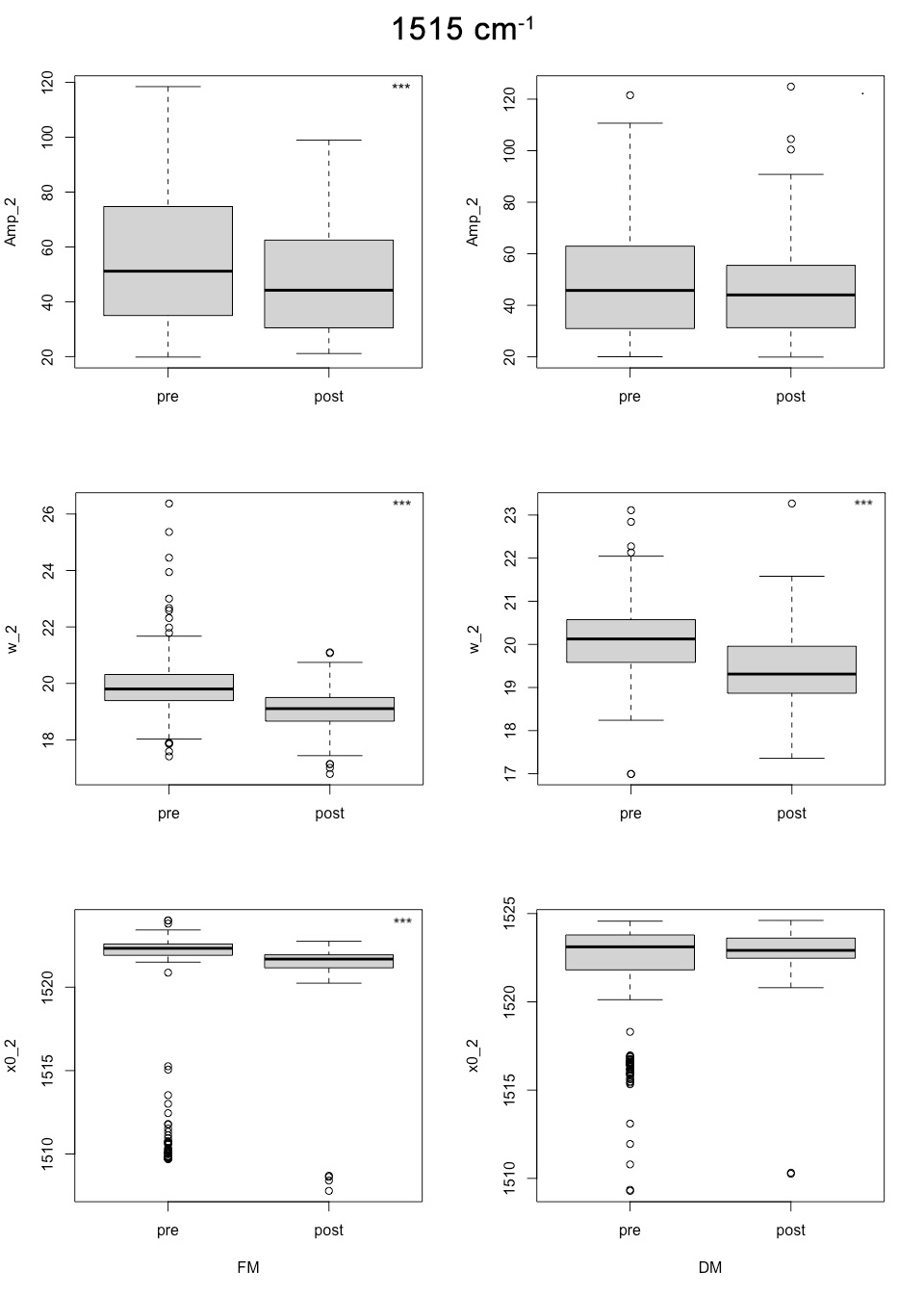


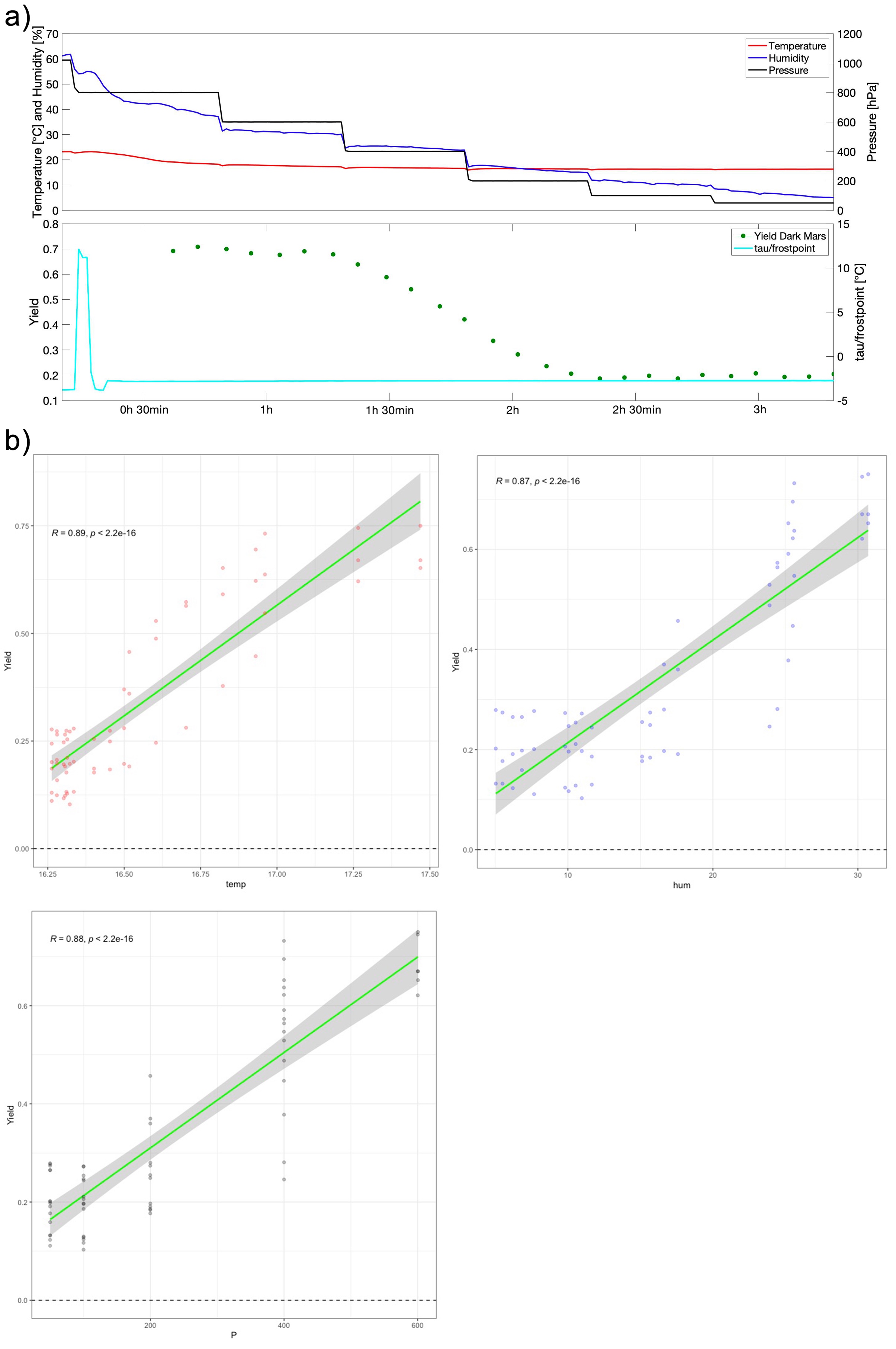


Figure S6 - a) Above. Temperature (red line), humidity (blue line) and pressure (black line) curves as performed in the PASLAB at DLR Berlin. Below. The green dots represent the Yield values of not irradiated samples and cyan line represents gas mixture humidity frost-point. b) Above on the left. Pearson’s correlation test plot between Yield and temperature indicated a significant correlation (R=0.89 p<0.001). Above on the right. Pearson’s correlation test plot between Yield and humidity indicated a significant correlation (R=0.87 p<0.001). On the left. Pearson’s correlation test plot between Yield and pressure indicated a significant correlation (R=0.88 p<0.001).

Figure S7 - Variation of the PSII (Y = F_V_/F_M_) efficiency before (pre_exp), after (post_exp) and 24 h, 48 h, 72 h, 96 h, 168 h and 192 h after the treatment. Solid lines = 30 days simulation; dashed lines = 7 days simulation. Blue line = external control, EC; magenta line = full Mars, FM; green line = dark Mars, DM. Error bars stands for confidence intervals. See Tab. S11 for 7-day simulation ANOVA results. See Tab. S12 for 30-days vs 7-days sim. recovery comparison ANOVA.


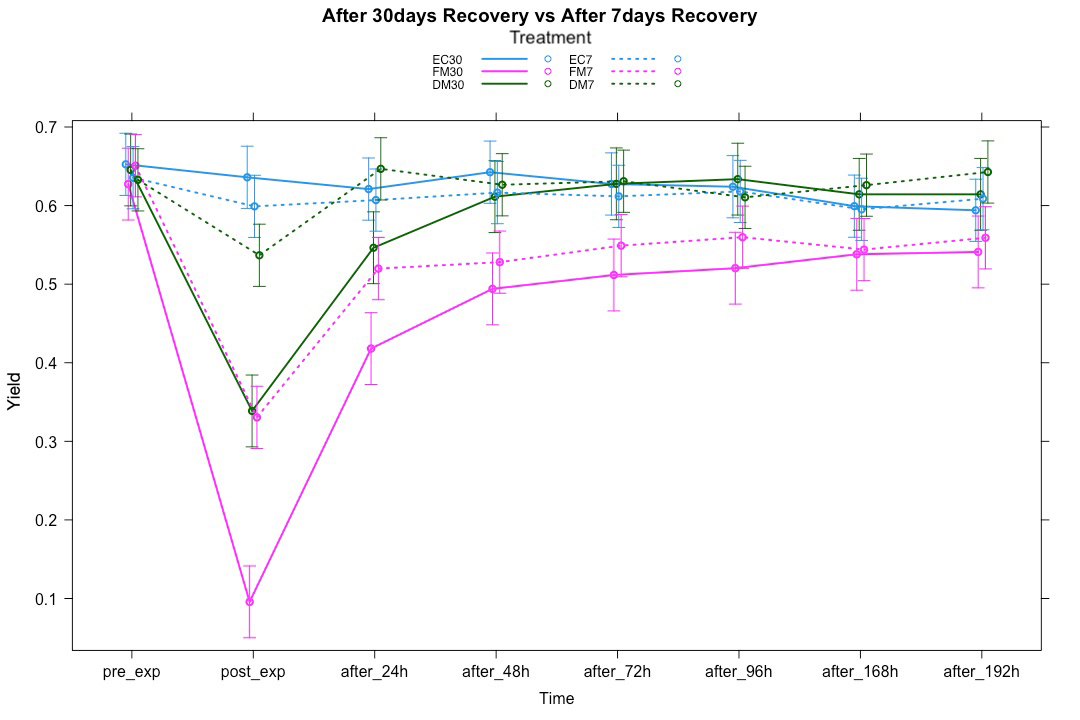


# **Supplementary Tables**

Table S1 - ANOVA type II Wald chi-square test's table for analysis of variance repeated measures for the relationship between Yield (Y(II) and F_V_/F_M_, depending on light or dark adaptation, related to day-night cycles in the Mars-like simulation) and treatments (FM and DM) over the exposure time period of the 30-days simulation. During the experiment, Yield values were measured every hour.

| **Factor** | **Chi Square** | **Num. Df** | ***p*-value** |
| --- | --- | --- | --- |
| Treatment | 17.502 | 1 | <0.001 *** |
| Time | 4733.030 | 665 | <0.001 *** |
| Treatment/Time | 883.339 | 665 | <0.001 *** |

Table S2 - Yield (Y = F_V_/F_M_) values before and after the exposure of the 30-days simulation. Four replicates (n) for each treatment. The values stand for Mean ± SD.

| **Treat-**  **-ment** | **pre_exp** | **post_exp** | **after_24h** | **after_48h** | **after_72h** | **after_96h** | **after_168h** | **after_192h** |
| --- | --- | --- | --- | --- | --- | --- | --- | --- |
| FM n=4 | 0.629 ± 0.016 | 0.096 ± 0.042 | 0.418 ± 0.023 | 0.494 ± 0.025 | 0.512 ± 0.019 | 0.520 ± 0.022 | 0.538 ± 0.011 | 0.541 ± 0.007 |
| DM n=4 | 0.627 ± 0.038 | 0.339 ± 0.083 | 0.546 ± 0.122 | 0.611 ± 0.083 | 0.628 ± 0.059 | 0.634 ± 0.051 | 0.614 ± 0.054 | 0.614 ± 0.058 |
| EC n=4 | 0.653 ± 0.020 | 0.636 ± 0.020 | 0.636 ± 0.077 | 0.643 ± 0.031 | 0.628 ± 0.037 | 0.624 ± 0.031 | 0.599 ± 0.039 | 0.594 ± 0.040 |

| **Factor** | **Chi Square** | **Num. Df** | ***p*-value** |
| --- | --- | --- | --- |
| Treatment | 25.784 | 2 | <0.001 *** |
| Time | 491.554 | 7 | <0.001 *** |
| Treatment/Time | 406.470 | 14 | <0.001 *** |

Table S3 - ANOVA type II Wald chi-square test's table for analysis of variance repeated measures for the relationship between Yield (Y = F_V_/F_M_) and treatments (FM, DM and EC) over times after the 30-days simulation. During the experiment, F_V_/F_M_ values were measured for each thallus before and after the exposure and 24 h, 48 h, 72 h, 96 h, 168 h and 192 h after the exposure.

Table S4 – F_0_ values before and after the exposure of the 30-days simulation. Four replicates (n) for each treatment. The values stand for Mean ± SD.

| **Treat-**  **-ment** | **pre_exp** | **post_exp** | **after_24h** | **after_48h** | **after_72h** | **after_96h** | **after_168h** | **after_192h** |
| --- | --- | --- | --- | --- | --- | --- | --- | --- |
| FM n=4 | 0.135 ± 0.018 | 0.136 ± 0.054 | 0.126 ± 0.054 | 0.107 ± 0.051 | 0.107 ± 0.056 | 0.106 ± 0.058 | 0.088 ± 0.035 | 0.081 ± 0.024 |
| DM n=4 | 0.141 ± 0.003 | 0.124 ± 0.010 | 0.094 ± 0.033 | 0.077 ± 0.032 | 0.085 ± 0.034 | 0.084 ± 0.033 | 0.092 ± 0.033 | 0.083 ± 0.038 |
| EC n=4 | 0.140 ± 0.008 | 0.141 ± 0.009 | 0.151 ± 0.021 | 0.131 ± 0.018 | 0.139 ± 0.016 | 0.132 ± 0.018 | 0.127 ± 0.034 | 0.122 ± 0.031 |

Table S5 - ANOVA type II Wald chi-square test's table for analysis of variance repeated measures for the relationship between F_0_ and treatments (FM, DM and EC) over times after the 30-days simulation. During the experiment, F_0_ values were measured for each thallus before and after the exposure and 24 h, 48 h, 72 h, 96 h, 168 h and 192 h after the exposure.

| **Factor** | **Chi Square** | **Num. Df** | ***p*-value** |
| --- | --- | --- | --- |
| Treatment | 3.0529 | 2 | 0.21731 |
| Time | 57.4127 | 7 | <0.001 *** |
| Treatment/Time | 25.3760 | 14 | <0.05 * |

Table S6 - Carotenoid peaks features’ (Amp and w) mean pre-exp. and mean post-exp values retrieved with Lorentzian fit. The percentage decrease is reported on the last column of the table.

| **Treatment** | **Peak** | **Feature** | **Mean Pre** | **Mean Post** | **% decrease** |
| --- | --- | --- | --- | --- | --- |
| **FM** | **1,000 cm^-1^** | Amp | 18.0833 | 13.0209 | 28 |
|  |  | w | 17.3375 | 14.653 | 15 |
|  | **1,150 cm^-1^** | Amp | 51.1044 | 34.6703 | 32 |
|  |  | w | 19.7137 | 19.0906 | 3 |
|  | **1,515 cm^-1^** | Amp | 52.0571 | 38.1776 | 27 |
|  |  | w | 21.6192 | 19.0288 | 12 |
| **DM** | **1,000 cm^-1^** | Amp | 15.3174 | 14.2259 | 7 |
|  |  | w | 17.1869 | 14.5805 | 15 |
|  | **1,150 cm^-1^** | Amp | 42.9274 | 37.1698 | 13 |
|  |  | w | 19.6364 | 18.973 | 3 |
|  | **1,515 cm^-1^** | Amp | 44.1902 | 39.8111 | 10 |
|  |  | w | 21.3289 | 19.3909 | 9 |

Table S7 - Carotenoid peak positions’ (x) mean pre-exp. and mean post-exp values retrieved with Lorentzian fit. The peak shifting is reported on the last column of the table.

| **Treatment** | **Peak** | **Feature** | **Mean Pre (cm^-1^)** | **Mean Post (cm^-1^)** | **Peak shifting** $\boldsymbol{\Delta}\tilde{\boldsymbol{\nu}}$ **(cm^-1^)** |
| --- | --- | --- | --- | --- | --- |
| **FM** | **1,000 cm^-1^** | x | 1006.51 | 1005.67 | -0.84 |
|  | **1,150 cm^-1^** | x | 1156.98 | 1156.21 | -0.77 |
|  | **1,515 cm^-1^** | x | 1521.33 | 1521.51 | 0.18 |
| **DM** | **1,000 cm^-1^** | x | 1006.9 | 1007.64 | 0.74 |
|  | **1,150 cm^-1^** | x | 1157.72 | 1157.63 | -0.09 |
|  | **1,515 cm^-1^** | x | 1522.02 | 1522.71 | 0.69 |

Table S8 – One-way ANOVA test’s table for analysis of variance of the differences between the pre-exp. and post-exp. values of the carotenoid peaks’ features (Amp, w and x).

| **Treatment** | **Peak** | **Feature** | **Sum of Square** | **Mean Square** | **Num. Df** | **F value** | ***p*-value** |
| --- | --- | --- | --- | --- | --- | --- | --- |
| **FM** | **1,000 cm^-1^** | Amp | 874.0 | 874.0 | 1 | 18.16 | <0.001 *** |
|  |  | Amp_residuals | 28723 | 48.1 | 597 |  |  |
|  |  | w | 713.7 | 713.7 | 1 | 166.3 | <0.001 *** |
|  |  | w_residuals | 2562.6 | 4.3 | 597 |  |  |
|  |  | x | Not normally distributed | | | | |
|  | **1,150 cm^-1^** | Amp | 12598 | 12598 | 1 | 28.91 | <0.001 *** |
|  |  | Amp_residuals | 260121 | 436 | 597 |  |  |
|  |  | w | 5.3 | 5.258 | 1 | 4.004 | <0.05 * |
|  |  | w_residuals | 784.0 | 1.313 | 597 |  |  |
|  |  | x | Not normally distributed | | | | |
|  | **1,515 cm^-1^** | Amp | 12283 | 12283 | 1 | 23.35 | <0.001 *** |
|  |  | Amp_residuals | 314063 | 526 | 597 |  |  |
|  |  | w | 95.6 | 95.58 | 1 | 133 | <0.001 *** |
|  |  | w_residuals | 428.9 | 0.72 | 597 |  |  |
|  |  | x | Not normally distributed | | | | |
| **DM** | **1,000 cm^-1^** | Amp | 0 | 0.32 | 1 | 0.009 | 0.925 |
|  |  | Amp_residuals | 166630 | 35.23 | 472 |  |  |
|  |  | w | 360.3 | 360.3 | 1 | 106.3 | <0.001 *** |
|  |  | w_residuals | 1599.2 | 3.4 | 472 |  |  |
|  |  | x | 44.28 | 44.28 | 1 | 143.6 | <0.001 *** |
|  |  | x_residuals | 145.55 | 0.31 | 472 |  |  |
|  | **1,150 cm^-1^** | Amp | 998 | 997.7 | 1 | 3.318 | <0.1 **^.^** |
|  |  | Amp_residuals | 141920 | 300.7 | 472 |  |  |
|  |  | w | 9.3 | 9.266 | 1 | 4.879 | <0.05 * |
|  |  | w_residuals | 896.3 | 1.899 | 472 |  |  |
|  |  | x | 1.69 | 1.6930 | 1 | 4.542 | <0.05 * |
|  |  | x_residuals | 175.95 | 0.3728 | 472 |  |  |
|  | **1,515 cm^-1^** | Amp | 1383 | 1382.9 | 1 | 3,614 | <0.1 **^.^** |
|  |  | Amp_residuals | 180629 | 381.7 | 472 |  |  |
|  |  | w | 59.6 | 59.60 | 1 | 79.54 | <0.001 *** |
|  |  | w_residuals | 353.7 | 0.75 |  |  |  |
|  |  | x | Not normally distributed | | | | |

Table S9 - Non-parametric Kruskal-Wallis test performed - instead of ANOVA - on not normally distributed peak position data.

| **Treatment** | **Peak** | **Feature** | **Chi Square** | **Num. Df** | ***p*-value** |
| --- | --- | --- | --- | --- | --- |
| **FM** | **1,000 cm^-1^** | x | 252.21 | 1 | <0.001 *** |
|  | **1,150 cm^-1^** | x | 237.46 | 1 | <0.001 *** |
|  | **1,515 cm^-1^** | x | 141.72 | 1 | <0.001 *** |
| **DM** | **1,515 cm^-1^** | x | 0.12581 | 1 | 0.7228 |

Table S10 - Yield (Y = F_V_/F_M_) values before and after the exposure of the 7-days simulation. Four replicates (n) for each treatment. The values stand for Mean ± SD.

| **Treat-**  **-ment** | **pre_exp** | **post_exp** | **after_24h** | **after_48h** | **after_72h** | **after_96h** | **after_168h** | **after_192h** |
| --- | --- | --- | --- | --- | --- | --- | --- | --- |
| FM n=4 | 0.651 ± 0.033 | 0.331 ± 0.035 | 0.520 ± 0.015 | 0.528 ± 0.040 | 0.549 ± 0.031 | 0.560 ± 0.028 | 0.544 ± 0.032 | 0.559 ± 0.030 |
| DM n=4 | 0.633 ± 0.014 | 0.537 ± 0.059 | 0.647 ± 0.060 | 0.627 ± 0.023 | 0.631 ± 0.018 | 0.611 ± 0.061 | 0.626 ± 0.006 | 0.643 ± 0.032 |
| EC n=4 | 0.636 ± 0.032 | 0.599 ± 0.011 | 0.607 ± 0.012 | 0.617 ± 0.010 | 0.612 ± 0.007 | 0.618 ± 0.015 | 0.595 ± 0.022 | 0.609 ± 0.015 |

Table S11 - ANOVA type II Wald chi-square test's table for analysis of variance repeated measures for the relationship between Yield (Y = F_V_/F_M_) and treatments (FM, DM and EC) over times after the 7-days simulation. During the experiment, F_V_/F_M_ values were measured for each thallus before and after the exposure and 24 h, 48 h, 72 h, 96 h, 168 h and 192 h after the exposure.

| **Factor** | **Chi Square** | **Num. Df** | ***p*-value** |
| --- | --- | --- | --- |
| Treatment | 36.682 | 2 | <0.001 *** |
| Time | 312.324 | 7 | <0.001 *** |
| Treatment/Time | 223.438 | 14 | <0.001 *** |

Table S12 - ANOVA type II Wald chi-square test's table for analysis of variance repeated measures for the relationship between Yield (Y = F_V_/F_M_) and the two simulations’ (30 days vs 7 days) treatments (FM, DM and EC) over times. During the experiment, F_V_/F_M_ values were measured for each thallus before and after the exposure and 24 h, 48 h, 72 h, 96 h, 168 h and 192 h after the exposure.

| **Factor** | **Chi Square** | **Num. Df** | ***p*-value** |
| --- | --- | --- | --- |
| Treatment | 25.784 | 2 | <0.001 *** |
| Time | 491.554 | 7 | <0.001 *** |
| Treatment/Time | 406.470 | 14 | <0.001 *** |
